# Molecular Docking and Dynamics Simulation Study of *Hyrtios* *erectus* Isolated Scalarane Sesterterpenes as Potential SARS-CoV-2 Dual Target Inhibitors

**DOI:** 10.3390/biology10050389

**Published:** 2021-05-01

**Authors:** Sameh S. Elhady, Reda F. A. Abdelhameed, Rania T. Malatani, Abdulrahman M. Alahdal, Hanin A. Bogari, Ahmad J. Almalki, Khadijah A. Mohammad, Safwat A. Ahmed, Amgad I. M. Khedr, Khaled M. Darwish

**Affiliations:** 1 Department of Natural Products, Faculty of Pharmacy, King Abdulaziz University, Jeddah 21589, Saudi Arabia; 2 Department of Pharmacognosy, Faculty of Pharmacy, Suez Canal University, Ismailia 41522, Egypt; omarreda_70@yahoo.com (R.F.A.A.); safwat_aa@yahoo.com (S.A.A.); 3 Department of Pharmacy Practice, Faculty of Pharmacy, King Abdulaziz University, Jeddah 21589, Saudi Arabia; rmalatani@kau.edu.sa (R.T.M.); aalahdal2@hotmail.com (A.M.A.); hbogari@kau.edu.sa (H.A.B.); 4 Department of Pharmaceutical Chemistry, Faculty of Pharmacy, King Abdulaziz University, Jeddah 21589, Saudi Arabia; ajalmalki@kau.edu.sa (A.J.A.); kmohammad@kau.edu.sa (K.A.M.); 5 Department of Pharmacognosy, Faculty of Pharmacy, Port Said University, Port Said 42526, Egypt; a_mansour7799@yahoo.com; 6 Department of Pharmacognosy, Faculty of Pharmacy, Horus University-Egypt, New Damietta 34518, Egypt; 7 Department of Medicinal Chemistry, Faculty of Pharmacy, Suez Canal University, Ismailia 41522, Egypt

**Keywords:** Red Sea, *Hyrtios* *erectus*, scalarane sesterterpenes, COVID-19, main protease, Nsp15 endoribonuclease, dual-target docking, molecular dynamics simulation

## Abstract

**Simple Summary:**

The ongoing coronavirus disease-2019 (COVID-19) pandemic shows unprecedented challenges for the worldwide healthcare system. Despite the large clinical data concerning several therapeutic interventions and drug repurposing, results are still either preliminary or lacking adequate-clinical efficiency. Herein, different pharmacoinformatics approaches have been adopted such as molecular docking, ADME properties prediction and all-atom MD simulation to investigate several marine-derived scalarane derivatives as lead candidates against two of the major COVID-19 targets; main protease and Nsp15 endoribonuclease. The presented study clearly illustrates the fitness of the proposed scalarane molecules as promising clinical candidates for further development and future in-vitro/in-vivo studies against SARS-CoV-2.

**Abstract:**

Presently, the world is under the toll of pandemic coronavirus disease-2019 (COVID-19) outbreak caused by SARS-CoV-2. Lack of effective and safe therapeutics has stressed the scientific community for developing novel therapeutics capable of alleviating and stopping this pandemic. Within the presented study, molecular docking, ADME properties and all-atom molecular dynamic (MD) simulation, along with two standard antiviral agents (lopinavir and benzopurpurin-4B), were applied to investigate 15 scalaranes sesterterpenes natural compounds, purified from the Red Sea marine sponge *Hyrtios* *erectus*, as potential COVID-19 dual-target inhibitors. Following multi-step docking within COVID-19 main protease and Nsp15 endoribonuclease cavities, nine promising drug-like compounds exhibited higher docking scores as well as better interactions with the target’s crucial residues than those of reference ligands. Compounds **2**, **6**, **11**, and **15**, were predicted to simultaneously subdue the activity of the two COVID-19 targets. Dynamics behavior of the best-docked molecules, compounds **15** and **6**, within COVID-19 target pockets showed substantial stability of ligand-protein complexes as presented via several MD simulation parameters. Furthermore, calculated free-binding energies from MD simulation illustrated significant ligand’s binding affinity towards respective target pockets. All provided findings supported the utility of scalarane-based sesterterpenes, particularly compounds **15** and **6**, as promising lead candidates guiding the development of effective therapeutics against SARS-CoV-2.

## 1. Introduction

The ongoing coronavirus disease-2019 (COVID-19) pandemic imposes great influences on individuals and the economy as well as unprecedented challenges for the worldwide healthcare system [1]. Owing to its profound aggressiveness and global spread, high overall case fatality rates up to 4% are depicted for COVID-19, being much higher (10–40%) in severely affected patients [2,3,4]. Current COVID-19 management comprises aggressive supportive treatments for maintaining blood-oxygen saturation, electrolyte-water balance, and haemostasis [5]. Moreover, antibiotics, antiviral, and anti-inflammatory agents permit managing viral loads, secondary bacterial infection, and cytokine storm, respectively [6]. Ongoing clinical trials, including World Health Organisation-implemented SOLIDARITY, aimed for repurposing FDA-approved therapeutics [7]. Antiviral agents like ribavirin/interferon, remdesivir, and ritonavir/lopinavir with/without umifenovir were investigated for possessing clinical benefits throughout other respiratory infections such as SARS-Co-V, MERS-Co-V, HIV, influenza, and Ebola [8,9,10,11,12]. Nevertheless, limited COVID-19 clinical efficiency was depicted with ritonavir/lopinavir for possessing poor overall mortality reduction and beneficial advantages over the standard care within COID-19 severely infected hospitalized adult patients [13]. Moreover, ribavirin-interferon combination and other possible interferon combinations represented no clinical improvement for patients with mild-to-moderate COVID-19 infection [14].

Repurposing anti-inflammatory agents, including corticosteroids and the antimalarial drugs hydroxychloroquine and chloroquine, is still controversial. Using hydroxychloroquine with/without azithromycin showed significant cough remission plus reduction of body temperature and viral loads, however, small sample sizes and short follow-ups begat misleading efficacy [15]. On the other hand, a systematic review/meta-analysis study was represented by Peng et al. illustrating the safety and efficacy of chloroquine and analogues in China as well as the pharmacological considerations to avoid chloroquine’s potential risk in busy clinic particularly in elder, paediatrics, and pregnant women [16]. Despite the above evidence, there is still an unmet need for more efficient and safer therapeutic agents that are capable of alleviating and stopping the COVID-19 pandemic. For the benefit of advanced bioinformatics and cheminformatics as well as improved software generations and algorithms, computational approaches greatly contribute to the drug discovery and development being both cost and time effective [17]. Consequently, recent studies adopted virtual screening, molecular docking, or even drug repositioning techniques to unveil potential hits against COVID-19 relevant targets [18,19,20,21,22,23,24,25].

Several key targets have been the focus by many studies aiming to developed potential agents blocking the COVID-19 life cycle. The key target is the COVID-19 main protease (M-pro) that is essential for cleavage of the virus polyproteins to produce non-structural proteins as a part of its replicase-transcriptase complexes [26]. Being fundamental for COVID-19 replication, highly conserved across related viruses, and not present in humans, the COVID-19 M-pro arose as an attractive target to safely encounter the viral disease [27,28]. Another promising target is the COVID-19 endoribonuclease non-structural protein-15 (Nsp15 NendoU) where this uridylate-specific enzyme is associated with several RNA processing-related biological functions [29]. The enigmatic enzyme has been suggested to contribute to viral replication, interfere with the host innate immune responses, and conceal viral genome from host defenses through its degradation [30].

Enrolling the latter targets within structure-based computational approaches has revealed several antiviral hits either from natural resources or chemical libraries of approved drugs for their repurposing to encounter COVID-19 pandemic [21,31]. The *Salvadora persica* flavonoids were represented as potential inhibitors of COVID-19 M-pro, while the bioactive compounds from *Glycyrrhiza glabra* were identified as possible blockers of COVID-19 Nsp15 NendoU and S-protein [22,25]. Applying structure-based virtual screening using ZINC, TCMD 2009, and/or CHEMBL databases showed promising compounds against M-pro [32,33,34]. Likewise, investigating an in-house library of 123 antiviral drugs by virtual screening has identified potential compound against M-pro and 20-*O*-ribose methyltransferase [35]. For drug repurposing, studies investigated FDA-approved drugs for potential affinity towards several COVID-19 targets using different in-silico tools including consensus docking and combined virtual screening with supervised machine learning or molecular dynamics simulation [21,36,37].

**Figure 1:**
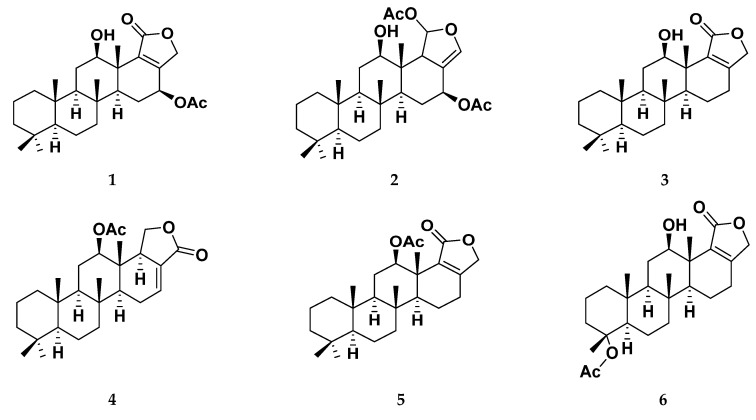

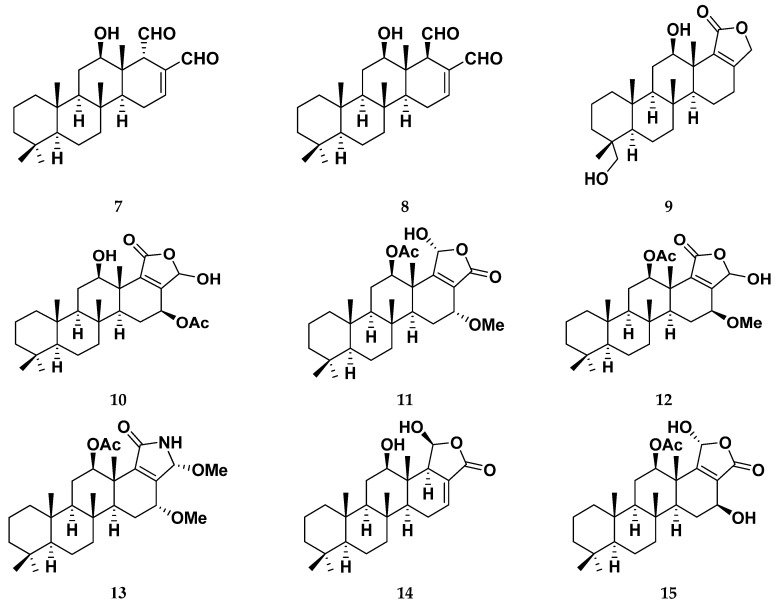
Structures of isolated scalaranes **1**–**15**.

In order to explore the less charted area of marine-based natural metabolites, here, within this presented manuscript, selected sesterterpenes analogues 1–15, isolated and identified from the Red Sea sponge *Hyrtios erectus* [38,39,40] (Figure 1), have been investigated for their potential COVID-19 multi-target inhibitory activity. The scalarane class of sesterterpenoids have been characterized and identified from the marine sponges of the order Dictyoceratida. The pharmacological activities of this class of sesterterpenes is of particular interest and display a variety of activities including antitubercular [40,41], anti-inflammatory [42,43], ichthyotoxic [44], antifeedant [45,46], antimicrobial [47,48], platelet-aggregation inhibitory effects [49,50] and antiproliferative [38,39,51,52,53,54,55,56,57,58]. In this study, the isolated scalarane-based metabolites were subjected to an in silico structure-based multitargeted molecular docking approach against the active sites of COVID-19 M-pro and Nsp15 NendoU proteins. Extensive investigation of ligand binding interactions with targets’ critical residues was performed to provide great insights regarding ligand’s structural preferentiality in relation to its favored target affinity. On the other hand, the validity and stability of the protein-ligand complex at the best docked hit were explored through all-atoms molecular dynamics simulation. Moreover, the binding free energy was also estimated from molecular dynamics trajectory to gain more insights regarding the type of ligand-protein affinity and interaction. Finally, the drug-likeness of the obtained hits was checked through calculating several molecular descriptors and pharmaceutically relevant properties being correlated to their expected pharmacokinetic properties (Absorption, Distribution, Metabolism, and Elimination; ADME) properties. Virtual calculation of these crucial properties provided valuable insights regarding the suitability of the obtained hits as future development of potential clinical candidates.

## 2. Materials and Methods

### 2.1. Marine Sponge Materials

The marine sample used in this study were collected from the Red Sea, Sharm el-Sheikh. The sample was frozen until investigation. The identification of sponge specimen was done by Dr. Rob van Soest as to be *Hyrtios erectus* of family: Thorectidae, order Dictyoceratida.

### 2.2. Isolation of Scalarane-Based Metabolites **1**–**15**

The Red Sea marine sponge *Hyrtios erectus* materials were collected using scuba diving and immediately frozen after collection until investigation. Compounds **1**–**15** (Figure 1) were isolated and purified using different chromatographic techniques as described previously [40]. In brief, the sponge samples were extracted with MeOH at room temperature and the extracts were concentrated under vacuum to yield the bioorganic crude extract. Using gradient elution on silica gel column chromatography, the total crude extract was fractionated to major nine fractions. The fractions 1–9 was chromatographed using different chromatographic techniques along with HPLC purification to obtain compounds **1**–**15**.

### 2.3. Multi-Target Docking Analysis of the Investigated Compounds

Effective in silico analysis of the 15 isolated scalarane sesterterpenes was performed on the adopted COVID-19 targets as compared to positive reference control agents using Molecular Operating Environment (MOE2019.01) software [59]. The docking protocol was performed on two stages, where the first is the preliminary stage where the MOE built-in virtual screening docking protocol was adopted for selecting the significant scalarane leads as compared to reference standard. This stage was performed for all 15 investigated compounds as well as one presumable positive reference ligand for each of the adopted targets. The COVID-19 clinically promising anti-viral agent, lopinavir, was assigned as reference control for M-pro, while benzopurpurin 4B was assigned for Nsp15 NendoU. Lopinavir, as a protease inhibitor, provided good results against SARS-CoV and significantly decreased the viral load in COVID-19 patients [8,60,61]. Lopinavir is in the top screened molecules from FDA approved drugs against COVID-19 [62]. The naphthalene-based azo compound, benzopurpurin 4B, showed great in vitro inhibition activity (0.2 μM) against the SARS-CoV Nsp15 endoribonuclease enzyme [63]. Recent study reported 88% sequence identity and 95% similarity for COVID-19 Nsp15 NendoU with its closest known homolog in SARS-CoV [29]. Such close similarity has rationalized the adoption of benzopurpurin 4B as a relevant reference ligand throughout the virtual docking screening approach.

**Figure 2:**
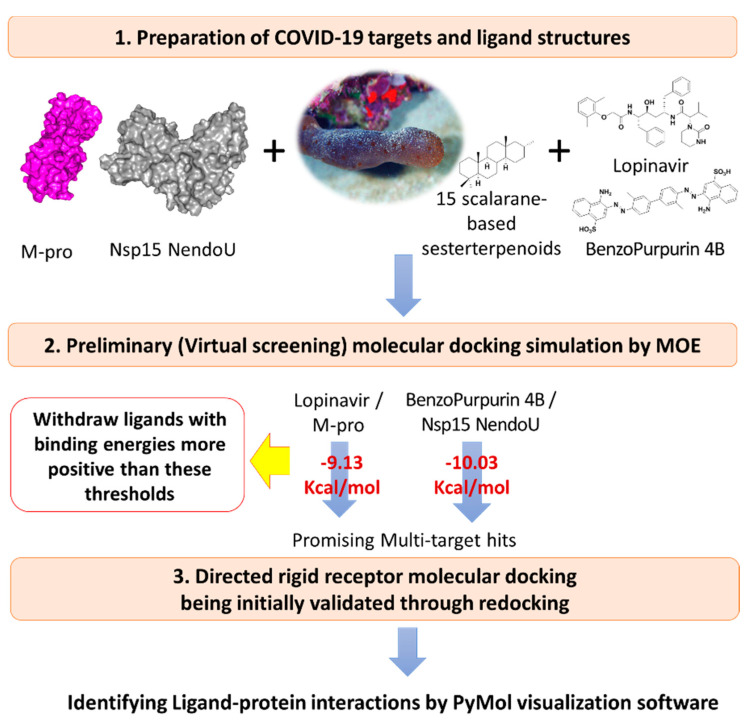
General scheme of the adopted multi-target molecular docking simulation of the selected scalarane-based sesterterpenoids against the adopted COVID-19 biological targets.

The second stage sophisticated docking protocol was performed to further validate the obtained docking results as well as obtaining valid docking poses for investigating the ligand-target binding interactions. This second stage docking is a more directed protocol which was conducted for the obtained hits at each target, where MOE rigid receptor docking protocol was assigned for M-pro and Nsp15 NendoU (Figure 2). Reported analysis of the M-pro binding site considered it of limited flexibility where S1′ and S1 subsites being either rigid or with slight changes at the end [21]. Moreover, a high superposition correlation (root-mean-square deviation; RMSD at C^α^ = 0.36 Å) between the M-pro apo-state and holo-state, with non-covalent inhibitor, (6m03 and 5r7z, respectively) indicated a non-presentable difference between both states. Additionally, the superposition correlation between M-pro in apo-state (PDB ID: 6m03) and in complex with covalent inhibitors (PDB ID: 6xho and PDB ID: 7jyC) ensures limited flexibility of the protein depicting low RMSD at C^α^ values of 0.52 Å or 0.29 Å, respectively. Interestingly, a RMSD at C^α^ = 0.44 Å was shown in the superposition correlation analysis between the M-pro bound to covalent (PDB ID: 5r7z) and non-covalent inhibitor (PDB ID: 7jyc). Similarly, binding of ligands within Nsp15 NendoU catalytic site was reported in non-significant protein conformational alterations, either at local or global levels, as compared to its apo structure (PDB ID: 6vww; RMSD at C^α^ = 0.29–0.39 Å for binding site important residues) [29]. The above findings suggested a non-relevant effect of the local ligand induced-fitting on M-pro or Nsp15 NendoU holo structures, at least within the macromolecule crystalline states [64]. Considering the above, preliminary docking was guided by the reported information of the target pocket and important residues responsible for ligand-protein interaction, thus, initial validation of this directed docking protocol was performed. Initial redocking of the co-crystallized ligand was performed within the target pocket adopting the similar protocol at the second directed docking stage for validating and ensuring the biological significance of the obtained docking poses and so the energies.

#### 2.3.1. Ligand Construction and Protein Preparation

All ligands were constructed via the MOE2019.01 (Chemical Computing Group^TM^, Quebec, Canada) builder tool and subsequently energy-minimized utilizing the MMFF partial charges and MMFF (modified) force field as being implemented with 2000 steps of conjugate-gradient method till a gradient of 1 × 10^−3^ Kcal/Å was reached [64,65,66]. Obtained ligand structures were saved in molecular database chemical file for incorporation within the molecular docking investigation. On the other hand, the atomic structures of the two investigated biological targets, M-pro (PDB ID: 5r7z) and Nsp15 NendoU (PDB ID: 6w01), were downloaded from the RCSB-Protein Data Bank. The adopted M-pro protein was the homo 2-mer-A2 of the COVID-19 protease enzyme in complex with an indole-based small molecule (Z1220452176; PDB ID: hwh), while the crystallized protein structure of Nsp15 NendoU is a Homo-6-mer -A6 in complex with citrate ions (PDB ID: cit). Downloaded PDB files were loaded into MOE for preparation through 3D-protonation as well as autocorrection for charges, atoms types, and connections. The 3D-protonation module permits identification of the acids/bases ionization states within Arg, Asp, Glu, Lys, and His residues while recognize the His imidazole tautomers and Asp/Glu carboxylic acids, at physiological pH (7), temperature of 300 K and 0.1 mol/L salt within implicit solvent at the Generalized-Born/Volumn-Integral implicit solvent model [11]. Typically, the module determines the protonation states from a titration curve and microstates populations of the protein atoms. Moreover, flipping protocol was adopted where atoms of Asp/Gln terminal amides and His imidazole were allowed to flip and the local hydrogen bond networks can be used to decide the flipped state of these groups [11]. Missing loops at the Nsp15 NendoU file was modeled using the MOE Loop modeler tool.

#### 2.3.2. Molecular Docking Protocol

Defining the binding site for each target was defined by MOE Alpha Site Finder and then refined to involve the crucial residues reported within the current literature. The defined M-pro and Nsp15 NendoU pocket was of 102 and 99 in size, respectively, where this value indicates the number of alpha spheres comprising this site. The MOE Site Finder tool depends on the geometric method where the relative positions and accessibility of the receptor atoms are considered along with a rough classification of chemical type. This uses alpha spheres which are the geometric features of the protein’s Voronoi diagram used to map out concave interaction [67]. An alpha sphere is a sphere that contacts four atoms on its boundary while containing no internal atoms. Additionally, crucial residues of the four subsites (S_1_′, S_1_, S_2_, and S_3_) at the M-pro binding site were included and considered relevant [28,68]. Regarding the Nsp15 endoribonuclease, a 20 Å radius region surrounding the crystallized citrate anion was considered as the active binding site comprising the six key conserved residues (His235, His250, Lys290, Thr341, Tyr343, and Ser294) across COVID-19, SARS-CoV, and MERS-CoV [29]. Throughout the adopted docking protocol, the ligand conformations were developed through the method of bond rotation, lodged within in the defined active site guided by a triangular-matching approach, and then conformations were ranked via the London_dG scores. The top ten docked poses (*n* = 10) were retained for subsequent refinement and then an energy minimization stage, within the target pocket, before they were rescored using Generalized-Born solvation-VI/Weighted-Surface Area_dG (GBVI/WSA) force field scorings. The latter MOE incorporated scoring system relies on Coulombic electrostatics using protein-ligand van der Waals score, current-loaded charges, exposure-weighted surface area, and solvation electrostatics [69]. Both the high docking energy and RMSD values (being kept below 2.0 Å) were considered for selecting the best docking pose for the designated ligand. Both analysis and visual inspection of the protein-ligand interactions for the obtained docking poses was achieved using the PyMol v2.0.6 Graphics System (Schrödinger^TM^, New York, NY, USA) [70]. Hydrophobic interactions were determined via the MOE ligand interactions tool, in addition to manual measurements done via the PyMol bond distance measurement tools.

### 2.4. Molecular Dynamics Simulations

For exploring the dynamic-nature of ligand-protein complex, the promising scalarane ligands in respect to each target were selected for 200 ns all-atoms molecular dynamics (MD) simulation using GROMACS software (GNU General Public License http://www.gromacs.org, access date, 1 November 2020) [71]. The CHARMM-GUI web server was used to generate the ligand-protein complex systems adopting the topology and parameters from the uploaded docked poses. Regarding the investigated ligands, the CHARMM force field parameters were used where they were automatically generated using the CHARMM General Force Field (CGenFF) program (ParamChem project; https://cgenff.umaryland.edu/, access date, 1 November 2020) [72]. The ligand-protein complex was centered within a 3D-cubic box, solvated using the TIP3P water model, while ionizable residues were assigned for standard ionization states under physiological pH (7). The systems were then neutralized with sufficient numbers of K ^+^ and Cl^−^ ions being added via Monte-Carlo ion-placing method [73], and finally simulated within periodic boundary conditions for eliminating any surface impacts. The CHARMM36 force field and constant number of particles, pressure, and 303.15 K temperature (NPT) ensemble were considered [25]. The prepared systems were subjected to one-step minimization and two-step equilibration stages to ensure that any bad or inappropriate contacts between the system components are resolved plus avoid any system errors interruptions during the MD runs. Along the two-stages, a 1000 kJ/mol force constant was implemented for restraining all heavy atoms to permit preservation of the original protein folding. The minimization step was proceeded through the steepest descent method for achieving a local energy minimum within the docked ligand-protein complexes permitting the resolve of any steric clashes or inappropriate geometry.

The minimization step was proceeded for 5000 steps (5 ps). For ensuring a reasonable starting structure, the equilibration procedure performed following the minimization step. Using the Berendsen thermostat for constant number of particles, volume, and temperature (NVT) ensemble, the first equilibration stage proceeded through a single-step protocol for 100,000 steps over a total duration of 100 ps [74]. Subsequently, the second-stage equilibration was proceeded for another 100 ps under constant number of particles, pressure (1 atm), and temperature (NPT) ensemble using Nose-Hoover thermostat and Parrinello-Rahman barostat [75]. The Verlet cut-off scheme, estimating a cut-off radius of 10 Å (1 nm), was used for the non-bonded interactions (Lennard-Jones and Coulomb potentials). The long-range electrostatic interactions were treated by the Particle-Mesh Ewald (PME) algorithm [76]. The new Linear Constraint Solver (LINCS) algorithm was used to constrain all covalent bonds including hydrogen atoms [77]. At this point, each system became minimized and well-equilibrated at the proper temperature (303.15 K) and ready for the 200 ns duration molecular dynamics runs. Three independent MD simulations were carried out for each ligand under constant number of particles, pressure (1 atm), and temperature (NPT) ensemble using Nose-Hoover thermostat and Parrinello-Rahman barostat [75], with an integration time step of 2 fs and without any restriction. The Verlet cut-off scheme was adopted estimating a cut-off radius of 10 Å (1 nm), for long-range interactions. Data analysis was performed using the Visual Molecular Dynamics 1.9.3 (VMD) package (University of Illinois at Urbana-Champaign, Champaign, IL, USA) and GROMACS, where three build-in trajectory tools, root-mean-square deviation (RMSD), RMS-Fluctuations (RMSF), and radius of gyration (Rg) were estimated to determine the molecular complex stability/validity in terms of conformation and performance [78]. Moreover, the binding-free energy was estimated by *g_mmpbsa* within GROMACS to gain more insights regarding the type of ligand-protein affinity and interaction as well as residue contribution within the binding free energy calculation [79].

### 2.5. Drug-Likeness Evaluation and In Silico ADME/TOX Prediction

Evaluating the drug-likeness propensity of proposed scalarane-based hits as well as their fitness as clinical candidates was performed by the means of Toxicity Estimation Software Tool^®^ version-4.2.1 (TEST^®^; Environmental Protection Agency, Washington, DC, USA) and QikProp^®^ V3.5 module (Schrödinger^TM^, New York, NY, USA). QikProp^®^ was run in normal mode, on Maestro^®^-GUI, for providing accurate prediction of various basic pharmaceutically relevant properties and physically significant descriptors being related to compound’s ADME/TOX properties [80]. Predicted basic physio-chemical properties included; the octanol/water partition coefficient (QPlogP_o/w_), aqueous solubility (QPlogS), apparent permeability across Caco-2-cells (gut-blood barrier model) for non-active transportation (QPPCaco), brain/blood partition coefficient (QPlogBB), apparent permeability through Madin-Darby canine kidney cells (blood-brain barrier mimicking model; QPPMDCK), human serum albumin binding (QPlogK_HSA_), and percent human oral absorption (% HOA) [81,82,83,84,85]. Compound’s toxicological profiles were evaluated through predicted IC_50_ for blockage of HERG K_v_11.1-channels (QPlogHERG) in addition to oral rat LD_50_ and AMES Mutagenicity testing via TEST^®^ through consensus method [86]. The latter two predicted properties represent the compound’s amount (mg/Kg) causing 50% rat death following oral administration and positive colony growth induction within any *Salmonella typhimurium* strain, respectively. Further drug-likeness assessment, regarding compound’s adherence to Lipinski’s rule of five (RO5), was performed through estimating specific molecular descriptors/properties including H-bond donors ≤ 5, H-bond acceptors ≤ 10, rotatable bonds < 5, mol_MW < 500, and QlogP_o/w_ < 5 [87]. Compounds with molecular descriptors/properties showing fewer, and preferably no, violations of both rules are more likely to be orally available and exhibit lower attrition rates throughout clinical trials [88,89].

## 3. Results and Discussion

### 3.1. Isolation and Identification of Scalarane Metabolites **1–15**

The marine sponge, *Hyrtios erectus*, materials were extracted with 100% methanol at room temperature and concentrated under reduced pressure to yield a residue (85 g). The resulted extract was subjected to fractionation using (*n*-hexane/CHCl_3_/MeOH) column chromatography gradient elution on silica gel. Successive chromatographic fractionation of the lipophilic fraction using column chromatography and final purification on HPLC yielded the pure metabolites **1–15**. Identification and structure elucidation of the isolated metabolites were deduced by study NMR spectroscopic data and comparison with literature data (Table 1) [38,39,40].

### 3.2. Multi-Target Molecular Docking and Structural-Based Activity Insights

**Figure 3:**
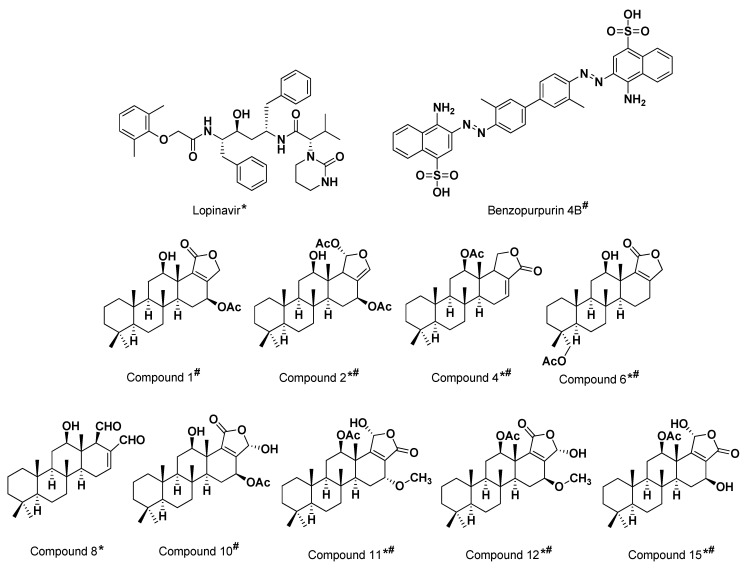
The 2D structural representation of the three positive reference ligands and promising hits targeting the two COVID-19 biological targets; * M-pro inhibitors and **^#^** Nsp15 NendoU inhibitors.

Results from the preliminary docking protocol showed that the reference ligands revealed binding energies of −9.1319 and −10.0317 Kcal/mol for the targets M-pro and Nsp15 NendoU, respectively. These reference binding energies were set as the threshold for selecting promising hits in term of highest negative binding energy. Interestingly, seven compounds on M-pro and eight ligands on Nsp15 NendoU, exhibited significant binding energies below those of their respective positive references (Table 1). Interestingly, four promising ligands **2**, **6**, **11** and **15** were found satisfactory for the whole two COVID-19 biological targets (Figure 3).

The validation of the sophisticated directed docking protocol within the co-crystallized M-pro (PDB ID: 5r7z; atomic resolution 1.59 Å) has depicted a root-mean-square deviation (RMSD) of 1.8201 Å for the redocked crystallized ligand (PDB ID: hwh). On the other hand, docking protocol validation at co-crystallized Nsp15 NendoU (PDB ID: 6w01; atomic resolution 1.90 Å) illustrated a RMSD of 1.1065 Å for the redocked crystallized ligand (PDB ID: cit) (Supplementary Materials, Figure S1). Clearly, depicting RMSD values below 2 Å indicates that both the adopted algorithms and parameters were sufficient for determining the best docking pose [90]. Thus, results out of the adopted directed docking protocol was confirmed valid, ensuring the biological significance of the obtained docking poses and so the energies.

### 3.3. Ligand/M-Pro Binding Interaction Analysis

**Figure 4:**
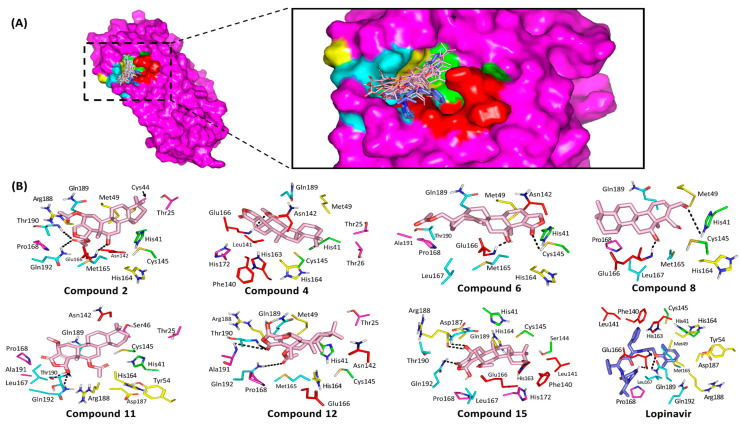
Ligand/M-pro binding interactions. (**A**) Surface rendition of M-pro (PDB ID: 5r7z), in magenta representation, with an overlay of investigated compounds (off-white lines) and lopinavir (slate blue sticks) at the enzyme active site (S1′, S1, S2, and S3 subsites colored as green, red, yellow, and cyan, respectively); (**B**) Predicted binding poses of docked ligands (sticks), only residues located within 5 Å radius of bound ligands are displayed (lines), colored according to their subsite location, and labeled with sequence number. Hydrogen bonding is depicted as black dashed lines.

After the directed rigid receptor docking protocol for the seven promising M-pro hits, significant binding interactions between these ligands and crucial M-pro-active site residues were predicted (Table 2). Similar to any protease enzyme, the M-pro binding site involves four important subsites, S_1_′, S_2_, S_3_ and S_4_, corresponding to their P_1_′, P_2_, P_3_ and P_4_ peptide substrate residues [28]. The docked ligands were positioned within the enzyme active site depicting favored contacts with the residues lining the four subsites (Figure 4A). A general conformation was predicted for all docked compounds where their polar *δ*-lactone rings face the S_3_ subsite while the distal part of the structure is anchored deep into the pocket at the S_1_′ and S_2_ subsites. This conformation was inverted for 6 which can be due to its specific 3α-acetyloxy group furnishing steric hindrance near the pocket subsites. Notably, limited access into the S1′ pocket was almost depicted for all docked compounds (Figure 4A). Several studies have revealed the crucial M-pro residues within each subsite influencing small molecule binding [21,22,26,68,91]. Hydrophobic contacts with Met165 and Gln189 from subsite S3 as well as His41, Met49, and Asp187 from S2 subsite serve as a hydrophobic grip to pin the ligands within the target pocket [21]. Despite being polar or even charged at physiological pH, both Gln189 and Asp187 depicted van der Waal interactions with their side chain Cβ and/or Cδ atoms with significant scalarane hydrophobic skeleton. On the other hand, both His41 and Cys145 represent the M-pro catalytic dyad at S1′ subsite where the interaction with these residues can greatly contribute to strong ligand-protein binding [68]. Only the docked ligands, **6** and **8** predicted polar interaction with Cyst145 or His41, respectively. Nevertheless, the compound **8**-His41 hydrogen bond pair is quite distant (4.0 Å) with binding angle (106°) making the contribution of such interaction pair, within compound **8** docking score, is minimal. However, **6** exhibits extra bonding with His41 through π-H interaction via its *δ*-lactone ring which has been depicted favored at 3.5 Å distance (Figure 4B).

Anchoring compounds within M-pro pocket was also achieved through polar interactions with Glu166 of the S1 subsite. The Glu166 main chain nitrogen and/or oxygen were able to achieve relevant hydrogen bonding with the OH or acetyl group of all docked ligands with, except 12 and 15 (Supplementary Materials, Table S1). The depicted hydrogen bond interactions with Glu166 mainchain was depicted within the optimum range, 1.9–3.2 Å and 132–159°, except for compound 11 where distant polar interaction and moderate angle of approach was shown (4.0 Å and 106°). The latter observations suggest a key role of Glu166 with the ligand/M-pro binding. Other S_1_ subsite residues (Phe140, Leu141, and His163) could not afford close proximity or polar interaction with any of the docked scalarane-based ligands suggesting a much less significant role for them. Regarding the reference ligand, lopinavir, an extended conformation was predicted where its tetrahydropyrimidine and 2,6-dimethylphenoxy terminal are directed towards the S_3_ and S_2_ subsites, respectively. Other identified important residues, such as Thr24, Thr25, Thr26, Pro168, His172, Phe185 and Ala191, depicted interactions with some of the docked scalarane-based ligands [26,68]. Throughout different orientation, lopinavir showed favored anchoring at S_1_ site with its benzyl substituent making hydrophobic contacts with Phe140, Leu141, Asn142, and His163. Anchoring of lopinavir within the active site was also assisted by polar interactions with both the carbonyl and NH of Glu166 mainchain as well as Gln189 sidechain via its amidic oxygen, tetrahydropyrimidine NH and phenoxy scaffolds. Nevertheless, the latter lopinavir-Gln189 interaction is compromised with the far distance (4.06 Å) between the hydrogen bond donor of lopinavir amide linker and Gln189 sidechain carbonyl, despite the relevant hydrogen bond angle (120°).

### 3.4. Ligand/Nsp15 NendoU Binding Interaction Analysis

**Figure 5:**
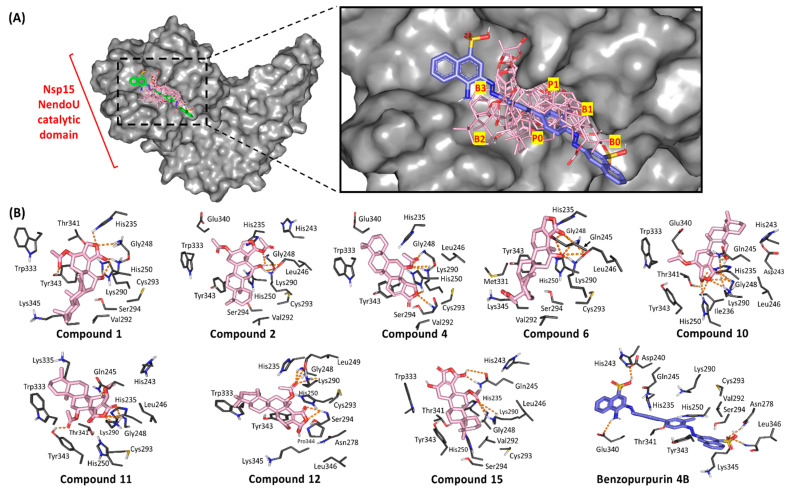
Ligand/Nsp15 NendoU binding interactions. (**A**) Surface rendition of Nsp15 NendoU monomer (PDB ID: 6w01), in gray representation, with an overlay of investigated compounds (off-white lines) and benzopurpurin 4B (slate blue sticks) at the catalytic domain binding site (bases B1, B2, B3 and phosphoryl groups P0, P1 and P2; highlighted in yellow); (**B**) Predicted binding poses of docked ligands (sticks), only residues located within 5 Å radius of bound ligands are displayed (lines) and labeled with sequence number. Hydrogen bonding is depicted as orange dashed lines.

Throughout the directed docking simulation, all hit compounds were successfully anchored within the C-terminal catalytic domain active site of Nsp15 NendoU without inexplicable steric issues. Several research groups identified the crucial residues for RNA anchoring, uracil selectivity/recognition, and transphosphorylation based on comparative analysis of the enzyme active site between COVID-19, SARS-CoV, MERS-CoV, and eukaryotic RNase A [29,92]. Typically, the active site is shallow and large being subdivided into several distinct pockets (bases B1, B2, B3 and phosphoryl groups P0, P1 and P2) specially designated for binding and processing the RNA substrate (Figure 5). As a general observation, the eight top docking scored scalarane derivatives exhibited significant binding within several active site pockets. All ligands were electronically and sterically compatible with the topology of the Nsp15 catalytic site and adjacent spaces. Almost all ligands suggested an anchoring of their *δ*-lactone rings within the B3 pocket (Figure 5A). However, only **4** and **12** illustrated an inversed docking orientation with preferential posing of their polar rings at the P1 and B1 pockets, respectively. On the other hand, the reference azo dye ligand extendedly docked at all binding site pockets with further elongation beyond the terminal base pockets, B0 and B3 [63]. Such an extended binding mode was mostly related to the compound’s great rigidity and linearity imposed by its central bisphenyl scaffold and the flanked naphthyldiazeno groups on both sides. The differential binding modes between the ligands and reference compound illustrate the complexity of Nsp15 NendoU binding site where it can accommodate both large and small ligands unless interactions with relevant amino acids is achieved.

Residues within the B1 site, Ser294 and Tyr343, govern the uracil recognition through polar/stacking forces. However, Trp333 at site B2 allows anchoring for RNA via π-stacking suggesting a significant force for ligands docking. The P1 site consists of the catalytic triad machinery, His235, His250, and Lys290, with the assistance from Gly248 main chain for pinning the substrate phosphoryl group. Both Thr341 and Asp240 were suggested significant for stabilizing His235 through direct hydrogen bonding or water-mediated polar interaction, respectively. The significance of the His-His-Lys triad and base-recognition Tyr343 has been established through mutational analysis illustrating reduced endoribonuclease activity [93,94,95]. Therefore, compounds exhibiting significant interactions with these residues are suggested to disrupt the RNA substrate recognition, anchoring, and/or cleavage based on the extent of the ligand-protein interaction. It is worth mentioning that, out of the six key conserved residues, only Lys290 in COVID-19 Nsp15 NendoU exhibits a non-conserved side chain conformation across SARS-CoV and MERS-CoV suggesting a key phylotypic-selectivity role for such residue.

Interestingly, relevant interactions have been depicted for the docked ligands with Nsp15 catalytic residues and vicinity (Table 3). All docked scalarane-based compounds managed to perform several polar interactions with important residues of P1 pocket through their *δ*-lactone rings and vicinal oxygen functionalities (Figure 5B). At least one hydrogen bonding has been depicted for all ligands with Lys290 side chain predominantly involved within nucleoside hydrolytic activity. The latter polar interactions were depicted optimum for compounds **1**, **2**, **4**, **6**, **10**, **11**, and **15** with bonding distance and angles being within the favored range (1.8–3.2 Å and 101–153°). However, the compound **12**-Lys290 polar interaction was compromised by its binding angle being below 100°, despite of the relevant proximity between the hydrogen bond donor and the heavy atom (2.5 Å). Anchoring of all ligands within P1 pocket was further stabilized through extended hydrogen bond network with Gly248 (1.7–2.9 Å and 111–163°) and/or Gln245 (1.7–3.4 Å and 113–147°) backbone chains. Despite the depicted polar interaction between compound **11** and Gln245 sidechain (<100°), the ligand showed strong stability at P1 pocket through optimum polar interaction with Gly248 (2.3 Å and 161°) favoring the adopted docking pose.

Interestingly, the docking poses of ligands (**1** and **10**) showed extra polar interaction with the indispensable catalytic histidine residues. Binding with either His235 or His250 has been predicted for **1** and **10** *δ*-lactone oxygen atoms, respectively. The depicted polar interactions were relevant at hydrogen bond distance/angle of 2.3 Å/111° and 2.8 Å/132° for compound **1** and **10**, respectively. On the other hand, compound **4** predicted weak hydrogen bond interaction with His250 being at far distance 3.6 Å with unfavored binding angle. Concerning the uracil-recognizing residues, suggested optimum polar interactions for only four ligands with Try343 hydroxyl group (acetyl moieties of **1** and **11**) or even NH of Ser294 main chain (**4** and **12**) was depicted (Supplementary Materials, Table S2). Moreover, the sesterterpenes **12** and **15** anchored at close proximity from Tyr343 permitting favorable π-hydrogen interaction being comparable to the substrate’s uridine moiety. Notably, Trp333 provided hydrophobic guidance for all docked ligands within the B2 base pocket. Based on the above findings, both compounds **12** and **15** exhibited comparable binding modes to the RNA substrate making them compatible with competitive inhibition and correlated to excellent docking scores (−10.9415 up to −11.2535 Kcal/mol, respectively). It is worth mentioning that the incorporated acetoxy function group within the compounds **12** and **15** structure predicted double polar interactions with significant pocket or catalytic residues, Gly248 and Lys290, being much favoured in compound **15** as compared to compound **12** (2.3–2.6 Å/144–163° versus 2.3–2.5 Å/97–135°, respectively). The latter observation may suggest the better docking scoring being assigned to compound **15** and in turn suggesting the significance of the acetoxy synthon for scalarane-based compound binding to Nsp15 NendoU pocket.

On the other hand, benzopurpurin 4B lacked any polar interaction with the catalytic/selectivity residues. Nevertheless, favorable hydrogen bonding with Asp240, His243, and Glu340 (site B3) as well as Asn278 and Leu346 (site B0), via its sulphonic and amino groups, allowed ligand successful anchoring across the whole binding site. Additionally, hydrophobic interactions with His235, Val292, Thr341, and Lys345 as well as bisphenyl π-hydrogen interaction with Tyr343, afforded a stable pocket accommodation affording high docking score affinity energy (−10.0997 Kcal/mol). These findings can reason the significant submicromolar in vitro NendoU inhibition activity of benzopurpurin 4B for the ligand’s extended occlusion of RNA-binding pocket. Although the scalarane-based hits showed less RNA-binding pocket occlusion, their extended polar interactions with catalytic/selectivity residues suggested significant blocking of enzyme catalytic machinery which can be equal-footed with that of benzopurpurin 4B. This can also provide rationalized explanation regarding the differential ligand-protein polar interaction pattern. The scalarane compounds interact majorly through being hydrogen bond acceptors, unlike Benzopupurin 4B which was introduced with equal contributions as hydrogen bond acceptor and donor. Therefore, the ability of the investigated scalarane compounds to form significant polar interaction with important pocket residues (Gly248, Gln245, His-His-Lys triad) which have been reported for catalysis, targeting, or fixing the ligands within the active pocket, which can rationalize their promising inhibition activity on NendoU Nsp15.

For providing more insights regarding the rational of differential binding between the investigated scalarane compounds and reference compounds, a pharmacophoric study was considered. Investigating the pharmacophoric features of scalarane-based hits illustrated significant findings. Generally, these ligands exhibit common pharmacophoric features involving a number of H-bond donners and acceptors, except for **4** where only H-bond acceptors were depicted (Supplementary Materials, Figure S2). These pharmacophores are confined to hydroxy, acetoxy, and aldehyde substituents or fused δ-lactone rings being relevant for polar interactions with crucial residues within the target pockets. Notably, lack of aromaticity and limited number of rotatable bonds, ranging from one and up to three, are the common features for these sesterterpenes. Suitable numbers of rotatable bonds permits relevant compound flexibility for adopting the best target pockets accommodation. However, excessive rotatable bonds become problematic, illustrating exponential conformation number and mostly correlated with decreased permeation rates [96]. Aromaticity, on the other hand, is significant for London dispersion forces and π-cation interactions with target proteins. Although investigated scalarane ligands lacked aromaticity, this was of no great influence on their binding as exhibited by better predicted docked energies than the aromatic references. Interestingly, the scalarane ligands possess significant lipophilic caged-like sesterterpene skeleton which can greatly contribute within the ligand-protein hydrophobic interaction and drug-pocket accommodation. Therefore, the lack of ligand’s aromaticity may be to some extent substituted by the presence of hydrophobic five-membered fused skeleton. Additionally, the extended polar interactions with crucial residues can further contribute with the ligand-target protein binding and complex stability. To further investigate such theories, extensive analysis of the ligand/target binding interactions was performed.

### 3.5. MD Simulation Analysis of Promising COVID-19 Multi-Target Inhibitor

MD simulation studies are considered effective for investigating the dynamic nature of ligand-target complex as well as relative stability. Moreover, MD simulations are especially useful for exploring the complex conformation space more efficiently than static image provided by molecular docking and mechanics energy minimization approaches [97]. The top docked ligands, with promising COVID-19 multi-target affinity, were subjected to 200-ns of MD simulation to understand the conformational changes of their drug/target complex throughout the interaction course. Both compound **15** and **6** in complex with Nsp15 NendoU and M-pro protein were proceeded for all-atom MD simulations. The latter selection was rationalized since both compound **6** and **15** have depicted the highest screening docking scores within the M-pro pocket. Moreover, across all high docking scored-ligands, only the promising scalarane compound **6** predicted significant strong interaction with the catalytic Cys145 (2.6 Å; 126°). Additionally, it showed strong hydrogen bonding with the important Glu166 ligand-directing residue (2.7 Å; 137°), besides the relevant non-polar contacts with hydrophobic pocket residues, particularly the π-H interaction (3.4 Å) with catalytic His41, suggesting its great stability within the pocket through the foreseeing MD simulation. Regarding selection of compound **15**-NendoU complex, the ligand depicted strong polar interactions with significant pocket residues, including two of the strongest hydrogen bonding with catalytic Lys290 (2.6 Å; 144°) and substrate-pinning assistant Gly248 (2.3 Å; 163°), relative to the other high docking-scored ligands. Additionally, compound **15** exhibited significant π-H interaction (3.4 Å) with B1 site hydrophobic residue, Tyr343, that was proven of its great role for uracil base recognition through stacking forces. The latter predicted compound **15**-Tyr343 hydrophobic interaction might infer greater ligand stability within the pocket through the foreseeing MD simulation. One different aspect, the investigation of compound **6**/NendoU complex through a MD simulation study would be beneficial to explore the dual affinity of the ligand towards the two COVID-19 biological targets.

#### 3.5.1. MD Analysis on M-Pro Target

**Figure 6:**
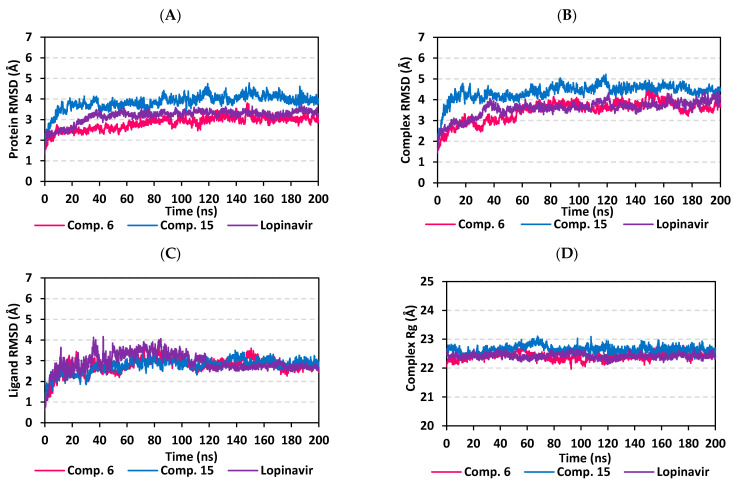
Stability analysis of the generated MD trajectories for both scalarane compounds and reference ligand in complex with M-pro protein throughout the 200 ns all-atom MD simulation. (**A**) protein C^α^ RMSD trajectories; (**B**) complex backbone RMSD trajectories; (**C**) sole ligand backbone RMSD trajectories; (**D**) complex Rg trajectories, across the simulation time (ns). The presented charts are the average representation of the triplicate MD simulation runs.

Throughout the 200 ns MD simulation run, the ligand/M-pro protein complexes showed great stability, with limited fluctuations, as being confirmed through the calculated RMSD, RMSF, and Rg. Generally, RMSD permits measuring the deviation of a molecule relative to a reference structure for providing a good indication for the stability and validity of the simulation protocol. High RMSD values for target infers instability and significant conformational changes [98], while the complex correlates to weaker ligand/target affinity being incapable to be contained within target’s active site throughout simulation period [99]. The calculated RMSD deviations for the M-pro proteins, with reference to its C^α^-atoms (RMSD C^α^), showed an overall typical behavior for MD simulations (Figure 6A). The protein RMSD trajectories increases over the initial frames due to the release of constrains at the beginning of the MD simulation run. Following 10 ns of the MD simulation start, stabilization was achieved for scalarane-based compounds where steady trajectories were depicted having the RMSD values levelled off at around 2.83 ± 0.32 Å and 3.86 ± 0.35 Å for compound **6** and **15**, respectively, till the end of MD simulation course. Concerning the lopinavir-bound protein, late stabilization was achieved being beyond the 30 ns. However, this protein managed to exhibit the steadiest trajectories following equilibration (3.29 ± 0.12 Å). The above depicted RMSD C^α^ tones infers that relaxation/equilibration stage, prior to the MD production, was sufficient enough as well as no further extension of the MD simulation beyond the 200-ns period.

For gaining more insights regarding the confinement of the scalarane-based ligand within the M-pro pocket during the MD simulation, the backbone RMSD fluctuation of the whole ligand-protein complex was monitored along the 200-ns simulation course (Figure 6B). Despite limited fluctuations, the binary complexes managed to reach their dynamic equilibration with respective RMSD plateau, beyond the 40 ns, indicating sufficient stabilization. Achieving early equilibration and the steadiest backbone RMSD trajectories, compound **15**/M-pro complex illustrated significant ligand accommodation within the M-pro binding site as compared to compound **6** and reference ligand, lopinavir. As a better descriptor for ligand/protein retainment within the target pocket, the sole ligand RMSDs, relative to the protein backbone, were monitored along the MD simulation runs (Figure 6C). Lower RMSD trajectories were assigned to the scalarane-based compounds as compared to lopinavir (Supplementary materials; Table S3 contains statistics of the triplicate simulation runs). This could be attributed to the lower extent of structural flexibility regarding the investigated scalaranes since these sesterterpenes incorporate less rotatable bonds as compared to the reference binder. Despite the differential RMSD tones at the initial MD simulation frames, the three binders managed to converge along the last 100 ns reaching to a final RMSD around 2.58 ± 0.11 Å.

Further stability analysis of the investigated ligand/M-pro complex was performed through estimating the Rg trajectories of complex entity (Figure 6D). The latter parameter permits the exploration of the complex rigidity and compactness across the MD trajectories. Typically, Rg is defined as the mass-weighted root-mean-square distance for group of atoms relative to their common mass center. Thus, the macromolecular structural alterations and overall dimensional changes can be explored by Rg throughout the MD simulation [100]. Within a valid simulation, the structure stability of a molecule is correlated to Rg reaching a plateau around the average. Beyond 60 ns simulation run, steadiest trajectories were assigned for compound **15** and lopinavir in complex with M-pro protein with average Rg trajectories of 22.65 ± 0.12 Å and 22.42 ± 0.08 Å, respectively, ensuring significant compactness and rigidity. On the other hand, a little lower average Rg value (22.41 ± 0.12 Å) was assigned for compound **6**/M-pro complex. The latter confers optimum structural compactness as favored inter- or intra-molecular interactions around this time frame. Interestingly, the three ligand/M-pro complex converge around similar Rg value (22.58 ± 0.13 Å), at the end of the MD simulation runs, ensuring the significant comparable stability and compactness of the three complexes.

It is worth mentioning that the ligand RMSDs were at lower values (~1.5-fold) than those of their respective proteins. All the above dynamic behaviors confirm significant ligand/pocket accommodation, successful complex stability and MD simulation convergence. However, further validation and monitoring of MD simulation convergence was performed via the principal component analysis (PCA) evaluating the protein’s collective dynamic motion/behavior from MD simulation trajectories. This approach depends on constructing and diagonalizing covariance matrix from the protein’s C^α^ atomic coordinates to capture strenuous atom motions using eigenvalues and eigenvectors [101]. Generally, the eigenvector of the covariance matrix correlates the overall atom’s motion direction, while eigenvalue represents the values of atom-wise contributions within motion. GROMACS “gmx_covar” command was used in constructing and diagonalizing the covariance matric, whereas, “gmx_anaeig” was for visualizing the most dominant modes (eigenvectors 1 and 2) besides calculating the trajectory coordinates/principal components overlap.

**Figure 7:**
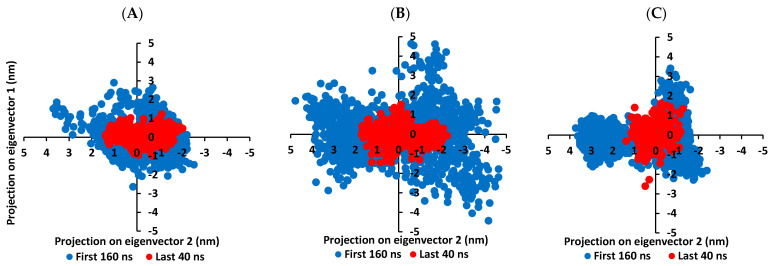
Projection of protein atoms in phase space along the first two dominant eigenvectors (eigenvector-1 and eigenvector-2). (**A**) compound **6**-bound M-pro protein; (**B**) compound **15**-bound M-pro protein; (**C**) lopinavir-bound M-pro protein. The PCA calculations were conducted cross initial 160 ns and last 40 ns MD simulation trajectories, having exhibiting differential expected structural stability and convergence. The presented charts are the average representation of the triplicate MD simulation runs.

With the corresponding eigenvalues providing an indication of the dynamic behavior and degree of fluctuations, lower covariance matrix trace values confer with minimal escalation within the collective protein motion and so denoting MD simulation convergence [102,103,104]. Applying PCA on the MD trajectories at the last 40 ns and comparing it with the initial MD simulation frames (first 160 ns) allowed efficient monitoring/validating the MD simulation convergence (Figure 7). As expected, lower covariance matrix average trace values were depicted at the last 40 ns for all investigated binders conferring successful protein convergence. This was obvious since the conformational space covered by the M-pro protein along the initial MD frames was wider. For compound **6**/M-pro protein an average trace value of 3.76 ± 0.66 nm^2^ and 5.51 ± 0.79 nm^2^ were assigned for the trajectories along last 40 ns and initial frames, respectively. Comparatively higher values were obtained with the protein’s atoms of the other binders (7.44 ± 0.74 nm^2^/9.78 ± 1.75 nm^2^ for compound **15**, and 5.57 ± 1.22 nm^2^/6.92 ± 0.45 nm^2^ for lopinavir at last 40 ns and initial frames, respectively). The latter dynamic behavior confers the higher comparative stability of compound **6**/M-pro protein, particularly over compound **15**. This came in good agreement with the significantly higher RMSDs of compound **15**-bound protein (Figure 6A). All the above findings ensure the higher stability of the protein atoms at the last 40 ns which in turn confer a validated convergence of the adopted MD simulation.

**Figure 8:**
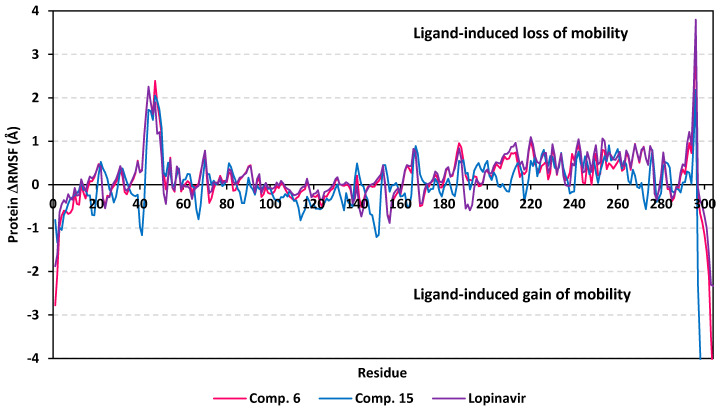
Analysis of ΔRMSF trajectories versus residue number for M-pro protein, in complex with both scalarane compounds and reference ligand, throughout the last 20 ns all-atom MD simulation. The ΔRMSF values, in reference to protein C^α^ atoms, were estimated cconsidering independent MD simulation of M-pro apo state against the holo states being complexed with the scalarane investigated ligands or reference lopinavir. The ΔRMSF trajectories are represented as a function of residue number (residues 1-to-306). The presented chart is the average representation of the triplicate MD simulation runs.

A final stability analysis was conducted where an estimated RMSF validation parameter highlights the contribution of individual amino acid residue within protein/ligand complex stability. Generally, RMSF evaluates the residue’s dynamic behavior (flexibility and movement) through explaining the mean deviation of each protein residue relative to its reference position over time. While more accurate, this validation parameter assesses the fluctuations of particular protein region from the average structure [105]. Within the presented manuscript, the difference root-mean-square fluctuation (ΔRMSF) was estimated for each ligand-bound protein relative to the apo state of COVID-19 M-pro protein. Adopting ΔRMSF cut-off value of 0.30 Å was relevant for estimating the significant change within structural movements, where residues with >0.30 ΔRMSF values were considered of decreased mobility [106]. Investigating the RMSF trajectories essentially execute for a trajectory region considered stable. Based on the above RMSD analysis, the C^α^ RMSF calculations were conducted at the last 20 ns of the MD simulation course. Typically, the free terminals residues and respective vicinal residues were shown to fluctuate with the highest negative ΔRMSF values in comparison to core residues (Figure 8). Interestingly, residues of the three ligand-protein complexes, within regions down towards the *N*-terminal of the M-pro protein exhibited higher fluctuation as compared to those flowing towards the other end. Notably, the terminal flexible residues are at regions being at distance of more than 13 Å from the active site residues, indicating the capability of the active site to accommodate bulkier inhibitors. Another general observation is that several distinct residue ranges, 41–52, 165–169/187–190, and 202–296, have exhibited significant immobility with the average ΔRMSF trajectories being above the 0.3 Å cut-off. Notably, the *N*-terminal vicinal residue range (290–296) exhibited the highest immobility profiles with ΔRMSF values up to 3.800 ± 0.07 Å. The latter dynamic behavior confers great impact of ligand binding on the stability of these *N*-terminal vicinal residues. On the other hand, a general trend of lower ΔRMSF values have been assigned for compound **15**-bound protein residues as compared with those for residues in complex with either compound **6** or lopinavir reference ligand. This was obvious across several protein residue ranges, particularly for those at 41–52 and 145–155 ranges. The latter ΔRMSF findings confer higher stability/immobility for compound **6** bound protein, being comparable to that of lopinavir, the thing that came in great agreement with the above Rg, RMSD, and PCA findings.

Regarding the flexibility of the pocket residues, almost all of the amino acids depicted significant ΔRMSF values above the cut-off threshold 0.3 Å with respect to their C^α^ atoms (Supplementary Materials; Table S4 contains statistics of the triplicate simulation runs). Residues comprising the S1′ sub-pocket exhibited the lowest of all ΔRMSF values, being below 0.3 Å, inferring the high flexibility indices for these residues. Nevertheless, only the catalytic His41 showed limited flexibility near the end of MD simulation with ΔRMSF trajectory of 0.35 ± 0.04 Å and 0.32 ± 0.09 Å for only compound **6** and lopinavir-bound proteins, respectively. The other catalytic residue, Cys145, exhibited significant mobility with all ΔRMSF being of negative values. This indicates the significant role of the ligand-His41 hydrogen bond pair over Cys145 for stabilizing the ligand-protein complex at the last 20 ns of MD simulation. On the other hand, the significant S2 sub-pocket residue Glu166 depicted higher ΔRMSF value within the three ligand-bound proteins (0.75 ± 0.11 Å, 0.58 ± 0.21 Å, 0.83 ± 0.04 Å for compound **6**, compound **15**, and lopinavir, respectively). This dynamic behavior infers its great contribution for ligand binding stability the thing that came in good agreement with the initial docking studies. Finally, all residues lining the S2 sub-pocket and most of the residues comprising the S3 one, particularly Met165, Leu167, and Gln189, depicted the highest significant immobility within the M-pro substrate binding site. Values of ΔRMSF for these significant immobile residues were 0.37 ± 0.05 Å to 1.55 ± 0.37 Å and 0.43 ± 0.07 to 0.74 ± 0.04 Å for S2 and S3 residues, respectively. Moreover, several S1′/S2/S3 vicinal residues depicted significant rigidity including, Pro39, Val42-Asp48, Leu50, Pro168, Phe185, and Val186 inferring stabilized ligand accommodation within these three respective protein sub-pockets. It is worth mentioning that this latter residue-wise immobility pattern was slightly less obvious with compound **15** than were a couple of S2 residues (Tyr54 and His164) which exhibited significant fluctuations. Nevertheless, the provided ΔRMSF findings illustrated the key role of S2 and S3 amino acids as well as vicinal residues for stabilizing saclarane-based compounds and lopinavir within the M-pro pocket. This came in high concordance with the predicted initial docking ligand-protein interactions. All presented Rg behaviours as well as findings from RMSD and ΔRMSF trajectories greatly imply sustained stability and compactness of the scalarane compound/M-pro investigated complexes across the all-atoms MD simulations.

#### 3.5.2. MD Analysis on Nsp15 NendoU Target

**Figure 9:**
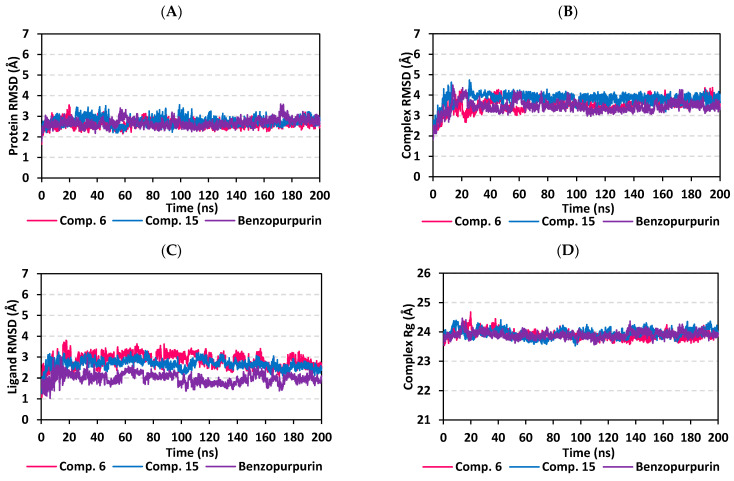
Stability analysis of the generated MD trajectories for both scalarane compounds and reference ligand in complex with Nsp15 NendoU protein throughout the 200 ns all-atom MD simulation. (**A**) protein C^α^ RMSD trajectories; (**B**) complex backbone RMSD trajectories; (**C**) sole ligand backbone RMSD trajectories; (**D**) complex Rg trajectories, across the simulation time (ns). The presented charts are the average representation of the triplicate MD simulation runs.

Findings of the MD analysis illustrated a better overall structure stability profile for Nsp15 NendoU complex, as compared to Mpro one, having less maxima and average deviations (Supplementary materials; Table S5 contains statistics of the triplicate simulation runs). For compound **15**-bound Nsp15 NendoU protein, the RMSD C^α^ tones smoothly shift from a value around 1.88 ± 0.24 Å at the simulation beginning to 2.93 ± 0.33 Å towards the end of MD runs (Figure 9A). Such behavior is expected for well-behaved simulations where macromolecules become stabilized over time. Regarding the Nsp15 NendoU proteins in complex with compound **6** and reference ligand, benzopurpurin 4B, limited fluctuation was also illustrated with significant converge reaching their respective own dynamic equilibrium following few initial nanoseconds (~5 ns). Following convergence, the RMSDs proceeded around comparable average RMSD C^α^ values of 2.62 ± 0.16 Å and 2.68 ± 0.20 Å for compound **6** and Benzopurpurin 4B, respectively. It is worth noting that the Nsp15 NendoU protein RMSD trajectories, across the three ligands, were of much steadier tones as compared to those of M-pro proteins for the same ligands. Depicting such steady dynamic behavior infer more preferential folding and stabilized secondary structure conformations of the bounded Nsp15 NendoU proteins, relative to M-pro proteins, across the 200-ns MD simulation.

Confinement of scalarene-based compounds within the Nsp15 RNA-binding site was correlated with the complex RMSD backbone tones (Figure 9B). Showing limited fluctuations, the backbone RMSD of ligand-protein complex increased gradually within first 15 ns nanoseconds where afterwards the RMSD levels-off around 3.83 ± 0.53 Å at the end of the MD simulation run. Managing to reach their respective dynamic equilibration with respective RMSD plateau over more than 100 ns indicates sufficient complex stabilization over the depicted MD simulation period. Following convergence, the steady RMSD C^α^ trajectories assigned for each complex (average RMSDs 3.57 ± 0.24 Å, 4.00 ± 0.23 Å, and 3.45 ± 0.22 Å for compound **6**, compound **15**, benzopurpurin 4B, respectively) infer significant accommodation of ligand within Nsp15 NendoU pocket across the MD runs. Notably, the steadiest RMSD backbone trajectories were illustrated for compound **15**/NendoU complex suggesting limited ligand fluctuations within the target protein pocket. Therefore, the sole ligand RMSD tones were monitored across the whole MD simulation timeframe. Minimal fluctuations were assigned for each of the investigated ligand beyond the initial 20 ns MD simulation with average RMSD tones of 2.86 ± 0.29 Å, 2.67 ± 0.29 Å, and 1.98 ± 0.22 Å for compound **6**, compound **15**, and reference ligand, respectively (Figure 9C). It is worth mentioning that the ligand RMSD tones were below those of their respective proteins, along most of the MD simulation, the thing that ensures significant ligand/pocket accommodation, successful complex stability and MD simulation convergence. Interestingly, the complex and ligand RMSD findings were highly correlated with respective complex Rg trajectories exhibited consistent tones without large fluctuations throughout the MD runs (Figure 9D). The Rg trajectories proceeded around an average value for each respective complex (23.90 ± 0.14 Å, 23.95 ± 0.15 Å, and 23.91 ± 0.11 Å for compound **6**, compound **15**, benzopurpurin 4B, respectively) till the end of the MD simulation run. Depicting these close complex Rg tones for each of the three investigated ligands infer comparable compactness and ligand confinement within the RNA-binding site.

**Figure 10:**
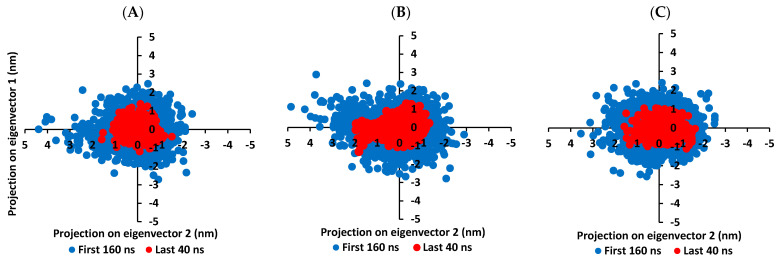
Projection of protein atoms in phase space along the first two dominant eigenvectors (eigenvector-1 and eigenvector-2). (**A**) compound **6**-bound Nsp15 NendoU protein; (**B**) compound **15**-bound Nsp15 NendoU protein; (**C**) benzopurpurin-bound Nsp15 NendoU protein. The PCA calculations were conducted cross initial 160 ns and last 40 ns MD simulation trajectories, having exhibiting differential expected structural stability and convergence. The presented charts are the average representation of the triplicate MD simulation runs.

Convergence within MD simulation was monitored and validated via the PCA approach relying on comparative analysis of the collective protein motion escalations performed on the MD trajectories at the initial 160 ns and last 40 ns timelines. Findings within Figure 10 illustrate lower average trace value of the covariance matrix within the last 40 ns as compared to first 160 ns MD simulation trajectories. Presenting such dynamic behavior confers successful convergence of the bounded proteins. Along the last 40 ns frames, the average trace values of the covariance matrix were comparable across the three ligands; 4.13 ± 0.49 nm^2^, 4.32 ± 0.60 nm^2^, and 4.10 ± 0.51 nm^2^ for compound **6**, compound **15**, and benzopurpurin 4B, respectively. These trace values were significantly lower (*p* < 0.05) than their respective values within the first MD trajectories (5.23 ± 0.78 nm^2^, 5.47 ± 0.13 nm^2^, and 5.30 ± 0.40 nm^2^, respectively). Interestingly, tighter trace values and more concise data scattering were depicted for Nsp15 NendoU as compared to M-pro for the three investigated ligands. Thus, preferential binding of scalarane-based compounds towards Nsp15 NendoU protein over the protease target has been suggested.

**Figure 11:**
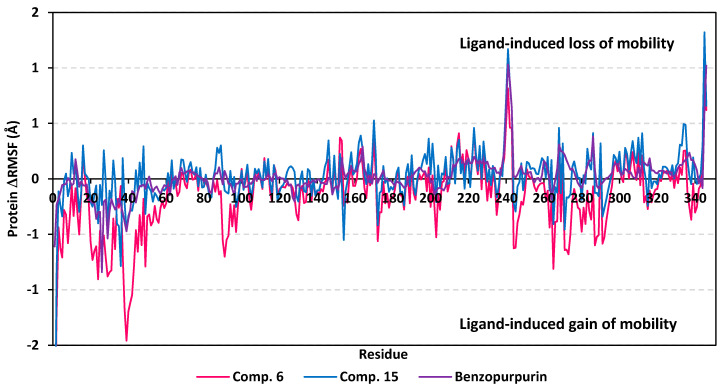
Analysis of ΔRMSF trajectories versus residue number for Nsp15 NendoU protein, in complex with both scalarane compounds and reference ligand, throughout the last 20 ns all-atom MD simulation. The ΔRMSF values, in reference to protein C^α^ atoms, were estimated considering independent MD simulation of Nsp15 NendoU apo state against the holo states being complexed with the scalarane investigated ligands or reference benzopurpurin 4B. The ΔRMSF trajectories are represented as a function of residue number (residues 1-to-346). The presented chart is the average representation of the triplicate MD simulation runs.

Moving towards the global protein residue flexibility, the C^α^ RMSF trajectories at the last 20 ns showed significant rigidity for great range of protein residues (Figure 11). The latter confirms the reported depicted rigidity of Nsp15 NendoU catalytic site, either at local or global levels, upon binding of ligands making the rational of adopting rigid-directing docking protocol acceptable [29]. It was interesting that the ΔRMSF values for the compound **6**-bound NendoU protein residues showed more negative values as compared to other ligand-bounded proteins. This can suggest an inferior compound **6**/protein complex stability as compared to the other saclarane and reference ligand. With a ΔRMSF cut-off of 0.3 Å, a specific region of residues (0-to-60) towards the *N*-terminal of Nsp15 NendoU protein exhibited the highest fluctuations with ΔRMSF tones down to ~−1.46 ± 0.25 Å for the three protein of the bounded ligands. Notably, these flexible residues are at region being very distant (>40 Å) from pocket residues, indicating the active site potentiality to harbor the larger-sized ligands. Further evaluation of active pocket RMSF trajectories illustrated differential flexibility among its constituting residues. On the other hand, terminal residues of the *C*-terminals as well as their vicinal residues depicted limited immobility with high ΔRMSF up to 1.32 ± 0.22 Å. The reason for such depicted stability is that these terminal residues are involved in the composition of the B0 subsite of the Nsp15 NendoU RNA-binding pocket where the three investigated ligands have predicted significant docking interactions.

Dissecting the inflexibility results for the key pocket residues, it was shown that residues of the B0 subsite exhibited significant inflexibility with ΔRMSF values being all positive (Supplementary Materials; Table S6 contains statistics of the triplicate simulation runs). Among the B0 residues, Lys345 and Leu346 exhibited the most noticed immobility (ΔRMSF 0.61 ± 0.59 to 1.32 ± 0.22 Å and 0.62 ± 0.70 to 1.02 ± 0.006 Å, respectively) across the three protein targets of the investigated ligands. On the contrarily, significant flexibility was assigned for most of the B1 subsite residues, except for the catalytic Lys290 amino acid. Only the proteins in complex scalarane-based compounds showed significant immobility for this positive-charged catalytic residue with ΔRMSF 0.31 ± 0.14 Å and 0.32 ± 0.10 Å for compound **6** and compound **15**, respectively. On the other hand, the other two catalytic residues, His235 and His250, depicted relevant flexibility across the three investigated complexes. The latter confer the superior role of Lys290, over the catalytic histidines, for stabilizing sacalarane-based compounds, particularly at the last 20 ns of the MD simulation.

Regarding the subsite B2 key residues, significant immobility was depicted for Trp333, the subsite B2 residue responsible for RNA substrate anchoring (ΔRMSF 0.33 ± 0.06 Å). This was only illustrated with compound **15**-bound Nsp15 NendoU the thing that can highlight the important role of the Trp333 π-hydrogen hydrophobic interaction for stabilizing scalarane within target pocket. Finally, Asp240 and His243 of the B3 subsite depicted significant immobility across the three ligands (ΔRMSF 0.50 ± 0.32 to 0.78 ± 0.11 Å and 0.46 ± 0.20 to 0.64 ± 0.13 Å, respectively) conferring their impact on the stability of the ligand-protein complex. Other than the pocket’s lining residues, several vicinal amino acids have depicted significant inflexibility including Leu215, Ile223, Tyr226, Leu228, Gly239, Phe241, Ser242, Leu332, Cys334, and Lys335 (Supplementary Materials; Table S6). These vicinal residues represent closer proximity towards the B2 and B3 subsites than the other pocket cavities. Additionally, the inflexibility profiles for these vicinal residues are highly correlated with compound **15** binding, yet least related to that of the reference ligand. The latter protein dynamic behavior highlights the more significant role of B3 residues and their vicinal amino acids for stabilizing the scalarane compounds, with higher preferentiality for compound **15**. In conclusion, all the above ΔRMSF findings infer great influence of scalarane binding for stabilizing the pocket residues suggesting a favored ligand-protein complex stability.

#### 3.5.3. Binding-Free Energy

The binding-free energy calculation, from the selected MD simulation trajectories, was performed to understand the nature of ligand-protein interaction as well as obtain more detailed information concerning the individual ligand contribution [107]. In this regard, the MD-directed Molecular Mechanics Poisson Boltzmann Surface Area (MM/PBSA) approach was implemented for binding free energy calculation, using the GROMACS *g_mmpbsa* tool, where higher negative binding energy explains more ligand affinity towards its respective target protein [79]. This approach can account for more accurate ligand-protein affinity as compared to static or even most sophisticated flexible molecular docking technique. The MM/PBSA is considered of comparable accuracy to the Free-Energy Perturbation approaches, yet with much smaller computational expenses [79]. Using the SASA-only model of the free-binding energy calculation as well as the single trajectory approach, representative frames were extracted/saved from the last 20 ns of MD simulation trajectories to be used for calculating each energy term across the three MD simulation runs and their average values (Table 4 and Supplementary Materials; Table S7).

To our delight, both promising scalarane-based ligands bound to COVID-19 targets, M-pro or Nsp15 NendoU, depicted significant free-binding (Table 4). Dissecting the obtained binding-free energy into its contributing energy terms has illustrated the high domination of the van der Waal interactions within the free-binding energy calculation as compared to that of the electrostatic energy term. Interestingly, the van der Waal domination was particularly obvious for ligands binding within the M-pro binding site, having the value of −132.92 ± 10.58, −106.70 ± 10.75, and −189.57 ± 12.57 kJ/mol for compound **6**, compound **15**, and lopinavir, respectively, as compared to theirs at NendoU RNA-binding site. This shows a higher hydrophobic nature of the COVID-19 M-pro sub-pockets as compared to those of Nsp15 NendoU. Additionally, the lower total non-polar interactions, as the summation of Δ*G*_vdw_ and Δ*G*_SASA_, for ligand/NendoU complex as compared to M-pro ones might be directly related to the M-pro larger pocket surface area. It is worth noting, that similar binding pattern was depicted for compound 6 and compound 15 across the docking and MD simulation study. Compound **6** exhibited more preferential free-binding energy as compared to compound **15** at the M-pro binding site, the thing that confirms the preliminary docking analysis. Such differential binding energies were in favor of compound **15** at the Nsp15 NendoU RNA-binding site which further came in good agreement with the docking study.

Across the three investigated ligand/M-pro complexes, compound **6** and **15** furnished the significant van der Waal energy contribution being just few 50 kJ/mol lower than that of the aromatic reference standard, lopinavir. The latter confirms the previous suggestion regarding the ability of the hydrophobic cage-like scalarane skeleton to overcompensate the lack of ligand’s aromaticity for ligand-target accommodation. For exhibiting more polar characters (hydrogen bond donors and acceptors), lopinavir exhibited higher electrostatic interaction energy and unfavored solvation energy (−31.56 ± 9.60 kJ/mol and 148.85 ± 16.24 kJ/mol, respectively) as compared to those of scalarane compounds. Therefore, significant scalarane ligand binding within the M-pro active pockets was illustrated better through the lower contribution of polar solvation energy terms since ligand-protein binding is a solvent-substitution process. Regarding the NendoU-bound complexes, benzopurpurin 4B exhibited the highest, yet comparable, van der Waal and electrostatic energy contributions (−111.03 ± 14.65 kJ/mol and −102.46 ± 14.83, respectively) which could be reasons for its respective highly hydrophobic conjugated naphthalene-based azo scaffold and polar double sulphonic acid functionalities. However, the latter hydrophilic groups were of double-bladed effect on the total free-binding energy contribution since this furnished the highest unfavored solvation energy (156.85 ± 24.82 kJ/mol). Thus, the final free-binding energy came in comparable values to those of the scalarene-based compounds, particularly for compound **15** (−72.82 ± 17.77 versus −49.73 ± 11.53 and −77.87 ± 19.49 kJ/mol). Notably, the higher binding free energy of compound **15**, over its other scalarane member, came in good agreement with the earlier RMSD, Rg, and ΔRMSF analysis regarding preferential stability for compound **15**/protein complex.

**Figure 12:**
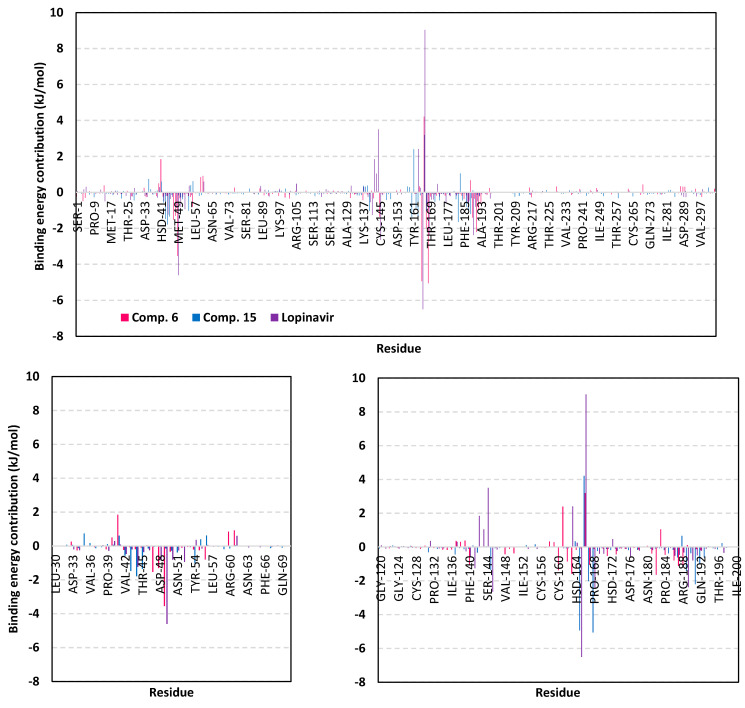
Binding-free energy decomposition illustrating the contribution of the protein target residues within the ligand/M-pro complexes binding-free energy calculation. This function was calculated through the *g_mmpbsa* tool in GROMACS. Lower panels are expanded versions of two designated residue regions (31–73 and 121–201) of the upper panel. The presented charts are the average representation of the triplicate MD simulation runs.

For gaining more insights regarding ligand-residues interactions, the binding-free energy decomposition within the *g_mmpbsa* module was utilized to identify the key residues involved within the obtained binding free energies [79]. Regarding the compound **6**/M-pro complexes (Figure 12), several residues have illustrated significant contributions within the calculated binding-free energy. Both Met165 (S3 sub-pocket) and its vicinal residue, Pro168, depicted the highest residue-binding energy contributions (−4.94 ± 0.12 and −5.06 ± 1.41 kJ/mol, respectively). Contribution of the key S2 sub-pocket residue, Glu166, was just below that of Met165 S3 sub-pocket residue.

Concerning, the S1′ sub-pocket catalytic dyad, both His41 and Cys145, showed significant contributions within the complex binding calculation with a higher preferentiality for Cys145. On the other hand, other residues of S3 sub-pocket showed significant contributions, particularly for Leu167, Gln189, and Gln192 as well as the vicinal residue, Ala191. Moving forwards for compound **15**/M-pro complex, lower value contributions by the pocket key residue was depicted as being expected from the furnished total free-binding energy. Significant residue-wise contribution was depicted with the S1 sub-pocket residues, Phe140, His163 and Glu166, ensuring the key role of these amino acids for compound **15** anchoring. The latter further highlights the important role of Glu166 for maintaining different members of scalarane-based ligands at M-pro pocket. On the other hand, both S2 and S3 sub-pocket residues were shown relevant for compound **15** binding since Tyr54, Asp187, and Arg188 at S2 sub-pocket as well as Leu167 at S3 sub-pocket, were assigned significant energy contributions. Additionally, several vicinal residues for S1′, S1, and S2 sub-pockets (Ile43, Cys44, Thr45, Tyr182, and Gly183) have illustrated significant hydrophobic residue-wise contributions as well as preferential accommodation of compound **15** within the latter sub-pockets.

Finally, residue-wise energy contribution for the M-pro protein in complex with lopinavir showed the most significant values for the pocket residues. The highest contribution was assigned for S2 Met49 and Glu166, S3 Met165, and S1 His163. The latter further infer the significant role of Glu166 to stabilize both scalarane and proteinomimetic ligands within the M-pro pocket. Notable contribution by Cys145 was depicted as superior over the other S1′ catalytic residue, His41 (−2.64 ± 1.29 versus 0.08 ± 1.71 kJ/mol). Several other residues showed moderate binding energy contributions including S1′ Ser144, S1 Leu141, and S3 Gln189. All the above findings can be correlated well with the reported inhibition activity of lopinavir being stably bounded to the key M-pro pocket residues.

**Figure 13:**
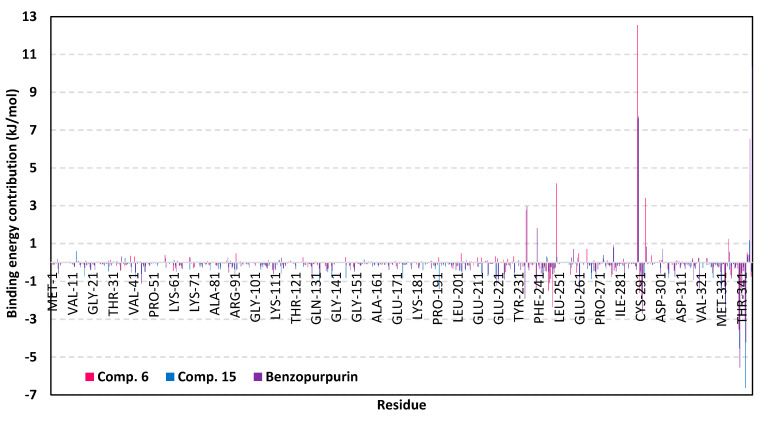

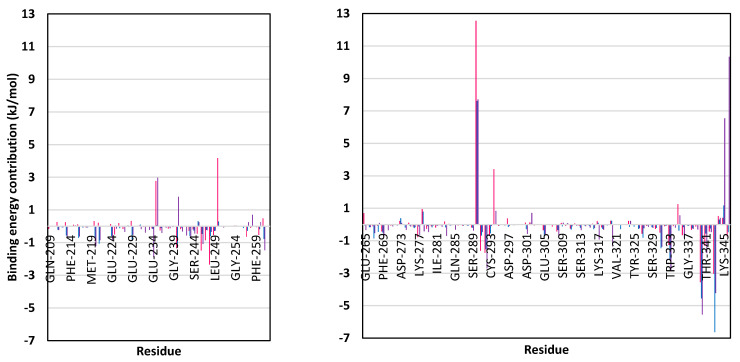
Binding-free energy decomposition illustrating the contribution of the protein target residues within the ligand-Nsp15 NendoU complex binding-free energy calculation. This function was calculated through the *g_mmpbsa* tool in GROMACS. Lower panels are expanded versions of two designated residue regions (209–263 and 266–346) of the upper panel. The presented charts are the average representation of the triplicate MD simulation runs.

Moving towards the Nsp15 NendoU-bounded ligand complexes, the binding-free energy decomposition highlighted the role of the RNA substrate anchoring residue, Trp333 at B2 subsite, for an overall ligand binding to Nsp15 NendoU active pocket (Figure 13). The highest contribution was assigned for the compound **15** and reference benzopurpurin 4B (−2.20 ± 1.29 and −2.80 ± 0.79 kJ/mol, respectively) ensuring the significance of the Trp333 π-H hydrophobic interaction for ligand anchoring. Moreover, the calculation illustrated the superiority of Tyr343 (−3.04 ± 2.71, −6.62 ± 2.25, and −4.22 ± 1.93 kJ/mol) over the other uracil recognition residue, Ser294 (3.41 ± 1.84, −0.03 ± 1.23, and 0.83 ± 0.89 kJ/mol), for anchoring squalane-based compounds as well as reference ligand, respectively, at the B1 site. The contribution of the catalytic triad was at higher share for Lys290/ligand binding as compared to those of His235 and His250. However, such energy contributions were unfavored for the total free-binding energy of compound **6** and benzopurpurin 4B as the residue-wise share illustrated high positive energy values. The latter highlights the negative impact of the central naphthalene-based azo scaffold for increasing the repulsive forces, while clearly explaining the depicted higher solvation energies for both compounds. Such unfavored repulsive forces were detrimental for compound **6** free-binding energy furnishing lower affinity as compared to compound **15** where the latter depicted less positive or even negative residue-wise energy contribution by the catalytic triad. On the other hand, the reference ligand managed to depict favored free-binding energy for the virtue of its balanced lipophilic/hydrophilic nature mediated by its aromatic and polar sulphonate scaffold providing high Δ*G*_van der Waal_ and Δ*G*_electrostatic_ energy contributions; −111.03 ± 14.65 and −102.46 ± 14.83 kJ/mol, respectively.

Notably, the B1/B2/B3 residues and their vicinal amino acids (Asp240, Leu246, Glu248, Cys291, Val292, Glu340, and/or Thr341) showed significant favored contribution for the free-binding energies of scalarane compounds. Again, the terminal residues, Lys345 and Leu346, showed the highest unfavored contribution with benzopurpurin 4B which could be related to increased repulsive forces with the ligand terminal naphthalene and sulphonic acid, respectively. This could ensure the double-bladed effect of sulphonic acid on benzopurpurin total free-binding energy contribution increasing the repulsive forces. It is worth noting that most of the binding energy contributions were confined with residues close to the *N*-terminal of Nsp15 NenondU which confirms the limited flexibility of these residues at the ΔRMSF trajectories (Section 3.5.2; Figure 11). Finally, all the above data contributed well with the favored binding of scalarane-based compounds within the Nsp15 NendoU RNA-binding site, particularly for compound **15**.

### 3.6. ADME/Tox Analysis and Values of Principal Molecular Descriptors

For investigating the fitness of marine scalarane-based hits, **6** and **15**, as promising clinical candidates, Lipinski’s RO5 and several crucial ADME descriptors/properties were adopted. Lipinski’s RO5 has been considered as the gold standard for drug-likeness and ADME assessment, yet not a strict criterion for natural products [108,109]. Interestingly, values of predicted descriptors/properties were found within the acceptable ranges provided by QikProp^®^ user manual for 95% of known drugs (Table 5). Both investigated hits predicted a moderate lipophilicity profile with balanced aqueous solubility at QPlogP and QPlogS values between (3.16 to 3.72) and (−5.73 to −5.77), respectively. Great adherence to the Lipinski’s RO5 has been predicted for both compound **6** and **15** showing no reported violation. Depicting the polar functionality (hydrogen bond acceptor and donor) below the Lipiniski’s RO5 threshold was beneficial for lowering the solvation energy for the scalarene-based compounds exhibiting significant free-binding energy being comparable to reference anti-viral agent, lopinavir (Table 4). Predicted compound’s permeation through different barrier models was significantly high illustrating QPPCaco and QPPMDCK values at (344.90 to 732.21 nm/s) and (156.55 to 353.21 nm/s), respectively. These values correlated well with high predicted % HOA (>90%), reaching up to 100% for compound **6**. Safety of investigated hits was suggested through depicting low predicted QPlogBB (−0.69 to −0.95) and high oral rat LD_50_ (>193.16 mg/Kg), inferring respective minimal influence on CNS and animal model mortality. Moreover, lack of impact on HERG K_v_11.1-channels and heart QT-prolongation was suggested for all investigated hits showing predicted QPlogHERG above −0.5. Compound mutagenicity was assigned negative for all compounds through TEST^®^ analysis. Regarding predicted human albumin protein-drug binding (QPlogK_HSA_), acceptable values were depicted for all hits, with the highest being for **2**, correlating to moderate drug-blood existence and drug accumulation. Based on the above findings, the investigated scalarane hits illustrated great drug-likeness and significant ADME/Tox profiles serving as promising clinical candidates for further development.

## 4. Conclusions

The COVID-19 pandemic is characterized by high mortality, morbidity, and wide-ranged severity. Despite the large clinical data concerning several therapeutic interventions and drug repurposing, results are still either preliminary or non-clinically efficient. Herein, adopting different pharmacoinformatics approaches such as molecular docking, ADME properties prediction, and all-atom MD simulation has revealed potential scalarane-based natural compounds as lead candidates against two of the major COVID-19 targets. Throughout the multi-step docking strategy, nine scalarane sesterterpenes isolated from the Red Sea marine sponge *Hyrtios erectus* were predicted higher docking scores on COVID-19 main protease and Nsp15 endoribonuclease as compared to anti-viral reference drugs. Compounds **2**, **6**, **11**, and **15**, exhibited significant docking scores inferring their capability to simultaneously subdue the activity of the two COVID-19 targets. Despite lacking aromatic pharmacophoric features, these promising scalarane compounds exhibited higher binding affinities with preferential polar binding interactions with the target’s crucial pocket residues.

All-atom MD simulation further validated the stability of the proposed binding modes of promising scalarane compounds, **6** and **15**, within their respective target’s cavities. The small RMSD, ΔRMSF, and Rg values, with minimal fluctuations, have been correlated to great stability and compactness of the respective ligand-protein complexes throughout the MD simulation runs. Additionally, ΔRMSF trajectories illustrated that the key residues involved within ligand-protein binding with the initial docking complex were of minimal flexibility at close to the MD simulation end. Furthermore, high negative binding-free energies for compounds **6** and **15** illustrated their significant affinity towards respective target molecule. Notably, predominance of the Van der Waal terms confirms the ability of the hydrophobic cage-like scalarane skeleton to overcompensate the lack of ligand’s aromaticity for ligand-target accommodation. Finally, the intensive ADME properties prediction using TEST^®^ and QikProp^®^ modules, illustrated great drug-likeness and significant ADME/Tox profiles of the two scalarane hits.

Future work concerning the exploration of enhanced sampling simulation approaches for studying the ligand binding/unbinding kinetics and thermodynamics would be adopted. The latter includes applications of alchemical free methods, Weighted Ensemble simulations, τ-RAMD simulations, scaled-MD and selectively scaled-MD simulations, weighted ensemble milestoning, transition path sampling, and Markov state analysis [110,111,112,113,114,115]. These approaches would be efficient for accelerating ligand dissociation and reliably predicting drug-likeness or drug efficacy through calculating drug residence times and binding/unbinding free energies. Moreover, the calculated pharmacophore features could be further applied to screen large libraries like ZINC using pharmer/ZINCPharmer in the future and apply ligand-based approaches to identify promising hits. As for the current time, the presented study clearly illustrates the fitness of the proposed scalarane molecules as promising clinical candidates for further development and future in vitro and in vivo studies against SARS-CoV-2.

## Figures and Tables

**Table 1:** Binding energies of docked compounds within the active site of M-pro (PDB ID: 5r7z) and Nsp15 NendoU (PDB ID: 6w01) of COVID-19 throughout the preliminary docking protocol.

Compound	Name	Binding Energy (Kcal/mol) ^a^	
		5r7z	6w01
1	Sesterstatin 7	−6.4842	**−10.6901**
2	Heteronemin	**−10.2889**	**−10.3251**
3	Scalarolide	−8.7294	−9.5520
4	12-epi-24-deoxyscalarin	**−10.3702**	**−10.6610**
5	Scalarolide acetate	−8.0121	−9.3353
6	19 acetylsesterstatin 3	**−10.8712**	**−10.3133**
7	12-deacetyl-12,18-di-epi-scalaradial	−7.0172	−7.8822
8	12-deacetyl-12-epi-scalaradial	**−9.5947**	−6.5206
9	Sesterstatin 3	−6.6789	−9.4241
10	12*β*,20α-dihydroxy-16*β*-acetoxy-17-scalaren-19,20-olide	−8.3400	**−10.6001**
11	12-O-acetyl-16-O-methylhyrtiolide	**−10.0880**	**−10.2102**
12	12*β*-acetoxy,16*β*-methoxy,20*α*-hydroxy-17-scalaren-19,20-olide	**−10.3784**	**−11.3058**
13	24*α*-methoxypetrosaspongia C	−6.0879	−8.2669
14	12-*O*-deacetyl-12,19-di-*epi*-scalarin	−8.1694	−8.6984
15	12*β*-acetoxy,16-*epi*-hyrtiolide	**−10.4134**	**−10.9658**
M-pro Reference	Lopinavir	−9.1319	–
NendoU Reference	Benzopurpurin 4B	–	−10.0317

^a^ Docking scores depicted as more negative values than compared to those of the control reference ligands were represented in bold.

**Table 2:** Parameters of binding interactions between the docked compounds and the active site of M-pro (PDB ID: 5r7z) throughout the directed rigid receptor docking protocol.

Compound	Docking Energy (Kcal/mol) ^a^		H-Bond Interactions	Hydrophobic Interactions	Sulphur-Dipole	π-Interaction	van der Waal with Side Chain Carbons
	Preliminary	Directed					
2	−10.2889	−12.0270	Glu166, Thr190, Gln192	Thr25, His41, Met49, Met165, Pro168	Met165 19*α*-acetyloxy	—	Gln189 (Cβ,Cδ)
4	−10.3702	−10.1306	Glu166	Thr25, Thr26, His41, Met49, Phe140, Leu141, Met165	—	—	Gln189 (Cβ,Cδ)
6	−10.8712	−12.7685	Cys145, Glu166	His41, Met49, Met165, Leu167, Pro168, Ala191	—	His41 (π-H)	Gln189 (Cβ,Cδ)
8	−9.5947	−10.8893	His41, Glu166	Met49, Met165, Leu167, Pro168	Met165 18*β*-aldehyde	—	Gln189 (Cβ,Cδ)
11	−10.0880	−11.5470	Glu166, Thr190, Gln192	Thr25, His41, Met49, Met165, Leu167, Pro168, Ala191	Met165 12*β*-acetyloxy	—	Gln189 (Cβ,Cδ) Asp187 (C*β*)
12	−10.3784	−11.0882	Thr190, Ala191, Gln192	Thr25, His41, Met49, Met165, Pro168, Ala191	—	—	Gln189 (Cβ,Cδ)
15	−10.4134	−12.4339	Arg188, Gln192	His41, Met49, Phe140, Leu141, Met165, Leu167, Pro168	—	—	Gln189 (Cβ,Cδ) Asp187 (C*β*)
Lopinavir	−9.1319	−10.0396	Glu166, Gln189	His41, Met49, Phe140, Leu141, Asn142, His163, Met165, Leu167, Pro168	—	—	Gln189 (Cβ,Cδ) Asp187 (C*β*)

^a^ MOE docking energy; Docking scores utilizing the scoring function assigned for the best-ranking poses and after refinement through the rescoring function of the GBVI/WSA dG incorporated within the MOE package.

**Table 3:** Parameters of binding interactions between the docked compounds and the active site of Nsp15 NendoU (PDB ID: 6w01) throughout the directed rigid receptor docking protocol.

Compound	Docking Energy (Kcal/mol) ^a^		H-Bond Interactions	Hydrophobic Interactions	π-Interactions
	Preliminary	Directed			
1	−10.6901	−10.7543	His235, Gly248, Lys290, Try343	Val292, Trp333, Thr341, Tyr343, Lys345	—
2	−10.3251	−11.7468	Leu246, Gly248, Lys290	Val292, Trp333, Thr341, Tyr343	—
4	−10.6610	−10.4221	Gly248, His250, Lys290, Ser294	Val292, Trp333, Thr341, Tyr343, Pro344	Trp333 (π-H)
6	−10.3133	−10.1971	Gln245, Leu246, Gly248, Lys290	Val292, Met331, Thr341, Trp333, Tyr343	—
10	−10.6001	−10.8170	Gln245, Gly248, His250, Lys290, Thr341	Ile236, His243, Leu246, Thr341, Trp333	—
11	−10.2102	−10.4656	Gln245, Gly248, Lys290, Tyr343	His243, Leu246, Trp333, Thr341, Lys335	—
12	−11.3058	−10.9415	Gly248, Lys290, Ser294	Val292, Trp333, Thr341, Pro344, Lys345, Leu346	Tyr343 (π-H)
15	−10.9658	−11.2535	Gln245, Gly248, Lys290	Val292, Trp333, Thr341, Leu246	Tyr343 (π-H)
Benzopurpurin 4B	−10.0317	−10.0997	Asp240, Asn278, Glu340, Leu346	His235, Val292, Tyr343, Thr341, Lys345	Tyr343 (H-π)

^a^ MOE docking energy; Docking scores utilizing the scoring function assigned for the best-ranking poses and after refinement through the rescoring function of the GBVI/WSA dG incorporated within the MOE package.

**Table 4:** Average of total binding-free energies and individual energy term (Δ*G* ± SE; across three independent MD simulation runs) concerning the promising sacalarane compounds and reference ligands within Nsp15 NendoU and/or M-pro protein binding sites.

Energy (kJ/mol ± SE)	M-Pro Complex			Nsp15 NendoU Complex		
	Comp. 6	Comp. 15	Lopinavir	Comp. 6	Comp. 15	Benzopurpurin 4B
Δ*G*_van der Waal_	−132.92 ± 10.58	−106.70 ± 10.75	−189.57 ± 12.57	−98.73 ± 7.84	−85.26 ± 12.10	−111.03 ± 14.65
Δ*G*_electrostatic_	−9.347 ± 9.51	−14.39 ± 9.37	−31.56 ± 9.60	−51.96 ± 7.96	−7.71 ± 5.32	−102.46 ± 14.83
Δ*G*_solvation_; Polar	81.32 ± 10.38	63.83 ± 18.13	148.85 ± 16.24	113.71 ± 12.92	25.36 ± 26.30	156.85 ± 24.82
Δ*G*_solvation_; SASA	−15.41 ± 0.77	−11.09 ± 1.47	−20.65 ± 1.11	−12.76 ± 0.80	−10.25 ± 1.35	−16.18 ± 1.66
Δ*G*_binding energy_	−76.37 ± 9.69	−68.3 ± 13.49	−92.93 ± 14.34	−49.73 ± 11.53	−77.87 ± 19.49	−72.82 ± 17.77

**Table 5:** Drug-likeness and predicted ADME descriptors/properties (with acceptable/recommended range ^a^) for scalarane-based hits.

Drug-Likeness/Predicted ADME Descriptors	Compound 6	Compound 15
QPlogP_o/w_ (−2.0 to 6.5)	3.72	3.16
QPlogS mol/dm^3^ (−6.5 to 0.5)	−5.73	−5.77
QPPCaco nm/sec (<25 poor >500 great)	732.21	344.90
QPPMDCK nm/sec (<25 poor >500 great)	353.21	156.55
QPlogBB (−3.0 to 1.2)	−0.69	−0.95
QPlogK_HSA_ (−1.5 to 1.5)	0.67	0.52
% HOA (<25% poor >80% great)	100	90.84
QPlogHERG (above −5.0)	−3.78	−3.52
Oral rat LD_50_ mg/Kg	193.16	628.19
AMES Mutagenicity test	Negative	Negative

^a^ Accepted/recommended ranges or values are reported from the QikProp^®^ user manual.

## Data Availability

Not applicable.

## Supplementary Materials

The following are available online at https://www.mdpi.com/article/10.3390/biology10050389/s1, Figure S1: Superimposing the crystallized (green sticks) and redocked (yellow sticks) ligands at (a) M-pro and (b) Nsp15 NendoU for validating the adopted directed docking protocol, Figure S2: The 3D structural representation of the promising scalarane-based hits (sticks) and reference ligands showing respective pharmacophoric features (mesh spheres) as well as projected virtual points (arrows). Features and directionality of H-bond donor, H-bond acceptor, or aromaticity are colored in violet, cyan, or orange, respectively, Table S1: Descriptive ligand/M-pro binding interactions through directed docking protocol. Table S2: Descriptive ligand/Nsp15 NendoU binding interactions through directed docking protocol. Table S3: Estimated RMSD and Rg parameter of investigated ligands and reference compound, lopinavir, complexed with M-pro proteins throughout the triplicate all-atom MD simulation. Table S4: Estimated ΔRMSF^a^ parameter of ligands/M-pro proteins across stable structure trajectories (last 20 ns). Table S5: Estimated RMSD and Rg parameter of investigated ligands and reference compound, Benzopurpurin 4B, complexed with Nsp15 NendoU proteins throughout the triplicate all-atom MD simulation. Table S6: Estimated ΔRMSF^a^ parameter of ligands/Nsp15 NendoU proteins across stable structure trajectories (last 20 ns). Table S7: Total binding-free energies and individual energy term (ΔG ± SD) of the promising scalarane-based compounds and reference ligands within Nsp15 NendoU and/or M-pro protein binding site, across the three independent MD simulation runs.
