# Supplementary material for: Molecular Docking and Dynamics Simulation Study of Hyrtios erectus Isolated Scalarane Sesterterpenes as Potential SARS-CoV-2 Dual Target Inhibitors
Source: Biology (Basel). 2021 May 1;10(5):389. doi: 10.3390/biology10050389 (PMC8147222; doi:10.3390/biology10050389)
Supplement: Supplementary file 1 [file biology-10-00389-s001.zip › biology-1119543-supplementary.pdf]

Molecular Docking and Dynamics Simulation Study of *Hyrtios erectus* Isolated Scalarane Sesterterpenes As Potential SARS-CoV-2 Dual Target Inhibitors

Sameh S. Elhady [1, †,*], Reda F. A. Abdelhameed [2,†], Rania T. Malatani [3], Abdulrahman M. Alahdal [3], Hanin A. Bogari [3], Ahmad J. Almalki [4], Khadijah A. Mohammad [4], Safwat A. Ahmed [2], Amgad I. M. Khedr [5,6] and Khaled M. Darwish [7,*]

[1] Department of Natural Products, Faculty of Pharmacy, King Abdulaziz University, Jeddah 21589, Saudi Arabia; ssahmed@kau.edu.sa
[2] Department of Pharmacognosy, Faculty of Pharmacy, Suez Canal University, Ismailia 41522, Egypt; omarreda_70@yahoo.com (R.F.A.A.); safwat_aa@yahoo.com (S.A.A.)
[3] Department of Pharmacy Practice, Faculty of Pharmacy, King Abdulaziz University, Jeddah 21589, Saudi Arabia; rmalatani@kau.edu.sa (R.T.M.); aalahdal2@hotmail.com (A.M.A.); hbogari@kau.edu.sa (H.A.B.)
[4] Department of Pharmaceutical Chemistry, Faculty of Pharmacy, King Abdulaziz University, Jeddah 21589, Saudi Arabia; ajalmalki@kau.edu.sa (A.J.A.); kmohammad@kau.edu.sa (K.A.M.)
[5] Department of Pharmacognosy, Faculty of Pharmacy, Port Said University, Port Said 42526, Egypt; a_mansour7799@yahoo.com
[6] Department of Pharmacognosy, Faculty of Pharmacy, Horus University-Egypt, New Damietta 34518, Egypt
[7] Department of Medicinal Chemistry, Faculty of Pharmacy, Suez Canal University, Ismailia 41522, Egypt
* Correspondence: ssahmed@kau.edu.sa (S.S.E.); khaled_darwish@pharm.suez.edu.eg (K.M.D.); Tel.: +966-544-512-552 (S.S.E); +20-100-5330-114 (K.M.D.)
† These authors equally contributed to this work.

Additional Experimental Detail

1) **Table S1.** Descriptive ligand/M-pro binding interactions through directed docking protocol.
2) **Table S2.** Descriptive ligand/Nsp15 NendoU binding interactions through directed docking protocol.
3) **Table S3.** Estimated RMSD and Rg parameter of investigated ligands and reference compound, lopinavir, complexed with M-pro proteins throughout the triplicate all-atom MD simulation.
4) **Table S4:** Estimated ΔRMSF[a] parameter of ligands/M-pro proteins across stable structure trajectories (last 20 ns).
5) **Table S5.** Estimated RMSD and Rg parameter of investigated ligands and reference compound, Benzopurpurin 4B, complexed with Nsp15 NendoU proteins throughout the triplicate all-atom MD simulation.
6) **Table S6:** Estimated ΔRMSF[a] parameter of ligands/Nsp15 NendoU proteins across stable structure trajectories (last 20 ns).
7) **Table S7.** Total binding-free energies and individual energy term ($\Delta G \pm$ SD) of the promising scalarane-based compounds and reference ligands within Nsp15 NendoU and/or M-pro protein binding site, across the three MD simulation runs.
8) **Figure S1.** Superimposing the crystallized (green sticks) and redocked (yellow sticks) ligands at (a) M-pro and (b) Nsp15 NendoU for validating the adopted directed docking protocol.
9) **Figure S2.** The 3D structural representation of the promising scalarane-based hits (sticks) and reference ligands showing respective pharmacophoric features (mesh spheres) as well as projected virtual points (arrows). Features and directionality of H-bond donor, H-bond acceptor, or aromaticity are colored in violet, cyan, or orange, respectively.

Table S1. Descriptive ligand/M-pro binding interactions through directed docking protocol

Compound	Ligand-target interaction description<br>[Type; Length (Å); Angle (°); Binding Residues]
**2**	H-bond ; 2.6 **Å** ; 132 ° ; Glu166 (main chain N**H** with 12*β*-**O**H)<br>H-bond ; 3.1 **Å** ; 131 ° ; Thr190 (main chain N**H** with 19*α*-OC(**O**)CH_3)<br>H-bond ; 3.0 **Å** ; 121 ° ; Gln192 (side chain N**H**$_2$ with 19*α*-OC(**O**)CH_3)<br>Sulphur-Dipole ; 3.3 **Å** ; Thr165 (side chain **S**CH_3 with 19*α*-**C**OOCH_3)
**4**	H-bond ; 3.2 **Å** ; 157 ° ; Glu166 (main chain N**H** with 12*β*-**O**C(O)CH_3)
**6**	H-bond ; 2.6 **Å** ; 126 ° ; Cys145 (side chain S**H** with 19-C=**O**)<br>H-bond ; 2.7 **Å** ; 137 ° ; Glu166 (main chain N**H** with 12*β*-**O**H)<br>π-H interaction ; 3.4 **Å** ; His41 (side chain)
**8**	H-bond ; 4.0 **Å** ; 106 ° ; His41 (side chain N**H** with 20-CH**O**)<br>H-bond ; 1.9 **Å** ; 159 ° ; Glu166 (main chain N**H** with 12*β*-**O**H)<br>Sulphur-Dipole ; 3.4 **Å** ; Thr165 (side chain **S**CH_3 with 20-CH**O**)
**11**	H-bond ; 4.0 **Å** ; 106 ° ; Glu166 (side chain N**H**$_2$ with 19-OH)<br>H-bond ; 1.9 **Å** ; 159 ° ; Thr190 (main chain N**H** with 12*β*-**O**H)<br>H-bond ; 1.9 **Å** ; 159 ° ; Gln192 (main chain N**H** with 12*β*-**O**H)<br>Sulphur-Dipole ; 3.4 **Å** ; Thr165 (side chain **S**CH_3 with 20-CH**O**)
**12**	H-bond ; 3.5 **Å** ; 101 ° ; Thr190 (main chain N**H** with 19-C=**O**)<br>H-bond ; 4.1 **Å** ; 93 ° ; Ala191 (main chain N**H** with 19-C=**O**)<br>H-bond ; 3.6 **Å** ; 105 ° ; Gln192 (side chain N**H** with 12*β*-OC(**O**)CH_3)
**15**	H-bond ; 3.5 **Å** ; 117 ° ; Arg188 (main chain N**H** with 16*β*-**O**H)<br>H-bond ; 2.4 **Å** ; 126 ° ; Arg188 (main chain C**O** with 16*β*-O**H**)<br>H-bond ; 2.3 **Å** ; 101 ° ; Gln192 (side chain N**H**$_2$ with 20-C=**O**)
**Lopinavir**	H-bond ; 1.9 **Å** ; 120 ° ; Glu166 (main chain N**H** with amide linker C=**O**)<br>H-bond ; 2.1 **Å** ; 108 ° ; Glu166 (main chain C**O** with pyrimidine N**H**)<br>H-bond ; 4.1 **Å** ; 132 ° ; Gln189 (side chain C**O** with amide linker N**H**)

Table S2. Descriptive ligand/Nsp15 NendoU binding interactions through directed docking protocol

**Compound**	**Ligand-target interaction description**<br>**[Type; Length (Å); Angle (°); Binding Residues]**
**1**	H-bond ; 2.3 **Å** ; 111 ° ; His235 (side chain N**H** with δ-lactone **O**)<br>H-bond ; 2.4 **Å** ; 135 ° ; Gly248 (main chain N**H** with δ-lactone **O**)<br>H-bond ; 1.8 **Å** ; 130 ° ; Lys290 (side chain N**H**$_2$ with 19-C=**O**)<br>H-bond ; 3.2 **Å** ; 101 ° ; Lys290 (side chain N**H**$_2$ with 12β-**O**H)<br>H-bond ; 1.9 **Å** ; 152 ° ; Try343 (side chain O**H** with 16β-OC(**O**)CH$_3$)
**2**	H-bond ; 2.3 **Å** ; 148 ° ; Leu246 (main chain C**O** with 12β-**O**H)<br>H-bond ; 1.7 **Å** ; 161 ° ; Gly248 (main chain N**H** with 12β-**O**H)<br>H-bond ; 2.4 **Å** ; 148 ° ; Lys290 (side chain N**H**H with 12β-**O**H)
**4**	H-bond ; 2.4 **Å** ; 111 ° ; Gly248 (side chain N**H** with 12β-**O**C(O)CH$_3$)<br>H-bond ; 3.6 **Å** ; 99 ° ; His250 (main chain N**H** with δ-lactone **O**)<br>H-bond ; 2.9 **Å** ; 153 ° ; Lys290 (side chain N**H**$_2$ with 12β-**O**C(**O**)CH$_3$)<br>H-bond ; 2.8 **Å** ; 174 ° ; Ser294 (main chain N**H** with δ-lactone **O**)<br>π-H interaction ; 3.8 **Å** ; Tyr343 (side chain)
**6**	H-bond ; 1.7 **Å** ; 141 ° ; Gln245 (side chain N**H**$_2$ with 19-C=**O**)<br>H-bond ; 2.7 **Å** ; 104 ° ; Leu246 (main chain C**O** with 12β-O**H**)<br>H-bond ; 2.9 **Å** ; 156 ° ; Gly248 (main chain N**H** with 12β-**O**H)<br>H-bond ; 2.9 **Å** ; 136 ° ; Gly248 (main chain N**H** with 19-C=**O**)<br>H-bond ; 1.9 **Å** ; 116 ° ; Lys290 (side chain N**H**$_2$ with 12β-**O**H)
**10**	H-bond ; 2.3 **Å** ; 113 ° ; Gln245 (side chain N**H**$_2$ with 19-C=**O**)<br>H-bond ; 2.1 **Å** ; 138 ° ; Gly248 (main chain N**H** with 19-C=**O**)<br>H-bond ; 2.8 **Å** ; 132 ° ; His250 (side chain N**H** with δ-lactone **O**)<br>H-bond ; 2.8 **Å** ; 148 ° ; Lys290 (side chain N**H**$_2$ with δ-lactone **O**)<br>H-bond ; 1.7 **Å** ; 144 ° ; Thr341 (side chain O**H** with 20-**O**H)
**11**	H-bond ; 2.1 **Å** ; 98 ° ; Gln245 (side chain N**H** with 16α-**O**CH$_3$)<br>H-bond ; 2.3 **Å** ; 161 ° ; Gly248 (main chain N**H** with δ-lactone **O**)<br>H-bond ; 2.4 **Å** ; 114 ° ; Lys290 (side chain N**H**$_2$ with 20-C=**O**)<br>H-bond ; 1.7 **Å** ; 132 ° ; Tyr343 (side chain O**H** with 12β-OC(**O**)CH$_3$)
**12**	H-bond ; 2.3 **Å** ; 135 ° ; Gly248 (main chain N**H** with 12β-OC(**O**)CH$_3$)<br>H-bond ; 2.5 **Å** ; 97 ° ; Lys290 (side chain N**H**$_2$ with 12β-OC(**O**)CH$_3$)<br>H-bond ; 2.3 **Å** ; 170 ° ; Ser294 (side chain N**H** with 19-C=**O**)<br>H-bond ; 2.6 **Å** ; 123 ° ; Ser294 (side chain N**H** with δ-lactone **O**)<br>π-H interaction ; 3.2 **Å** ; Tyr343 (side chain)
**15**	H-bond ; 3.4 **Å** ; 137 ° ; Gln245 (side chain C**O** with 19α-**O**H)<br>H-bond ; 1.8 **Å** ; 147 ° ; Gln245 (side chain N**H**H with 19α-**O**H)<br>H-bond ; 2.3 **Å** ; 163 ° ; Gly248 (main chain N**H** with 12β-OC(**O**)CH$_3$)<br>H-bond ; 2.6 **Å** ; 144 ° ; Lys290 (side chain N**H**H with 12β-OC(**O**)CH$_3$)<br>π-H interaction ; 3.2 **Å** ; Tyr343 (side chain)
**Benzopurpurin 4B**	H-bond ; 2.1 **Å** ; 152 ° ; Asp240 (side chain CO**O**H with SO$_2$O**H**)<br>H-bond ; 2.5 **Å** ; 164 ° ; Asn278 (side chain N**H**H with S**O**$_2$OH)<br>H-bond ; 2.6 **Å** ; 159 ° ; Glu340 (side chain CO**O**H with Ar-N**H**$_2$)<br>H-bond ; 2.6 **Å** ; 164 ° ; Leu346 (main chain N**H** with S**O**$_2$OH)<br>π-H interaction ; 3.1 **Å** ; Tyr343 (side chain)

Table S3. Estimated RMSD and Rg parameter of investigated ligands and reference compound, lopinavir, complexed with M-pro proteins throughout the triplicate all-atom MD simulation

		**Compound 6 / M-pro complex**				**Compound 15 / M-pro complex**				**Lopinavir / M-pro complex**			
		**RMSD protein** (Å)	**RMSD complex** (Å)	**RMSD ligand** (Å)	**Rg complex** (Å)	**RMSD protein** (Å)	**RMSD complex** (Å)	**RMSD ligand** (Å)	**Rg complex** (Å)	**RMSD protein** (Å)	**RMSD complex** (Å)	**RMSD ligand** (Å)	**Rg complex** (Å)
**1st MD run**	**Max.**	3.91	3.96	4.48	23.66	5.98	6.02	4.57	23.13	4.18	4.91	2.98	22.80
	**Min.**	1.52	1.59	0.53	21.71	1.45	1.48	0.69	21.61	1.39	1.50	0.52	21.87
	**Mean[a]**	2.58 ±0.24	2.63 ±0.24	2.94 ±0.46	22.41 ±0.27	4.30 ±0.55	4.30 ±0.49	2.87 ±0.42	22.20 ±0.25	3.10 ±0.38	3.41 ±0.42	2.21 ±0.30	22.31 ±0.14
**2nd MD run**	**Max.**	3.95	4.56	3.72	23.07	4.36	4.37	3.97	23.69	3.70	3.97	5.60	23.11
	**Min.**	1.40	1.43	0.95	21.98	1.80	1.81	1.15	22.39	1.31	1.34	0.88	22.02
	**Mean[a]**	2.51 ±0.34	3.09 ±0.50	2.52 ±0.39	22.40 ±0.16	3.14 ±0.35	3.64 ±0.34	2.74 ±0.3	22.95 ±0.16	2.70 ±0.41	3.14 ±0.32	3.80 ±0.60	22.54 ±0.14
**3rd MD run**	**Max.**	4.72	6.72	4.55	23.28	5.09	6.55	3.77	23.69	4.82	6.23	3.99	22.84
	**Min.**	1.43	1.48	0.49	21.91	1.54	1.56	0.86	21.93	1.48	1.46	0.43	21.93
	**Mean[a]**	3.42 ±0.59	4.70 ±0.94	2.79 ±0.77	22.43 ±0.30	4.22 ±0.46	5.19 ±0.53	2.69 ±0.50	22.78 ±0.24	3.74 ±0.56	4.17 ±0.76	2.74 ±0.44	22.41 ±0.13
**Average ± SD/SE**													
	**Max.[a]**	4.19 ±0.45	5.08 ±1.45	4.25 ±0.46	23.34 ±0.30	5.15 ±0.81	5.65 ±1.14	4.10 ±0.42	23.50 ±0.32	4.23 ±0.56	5.04 ±1.14	4.19 ±1.32	22.92 ±0.17
	**Min.[a]**	1.45 ±0.06	1.50 ±0.08	0.66 ±0.25	21.87 ±0.14	1.60 ±0.18	1.62 ±0.18	0.90 ±0.23	21.98 ±0.40	1.39 ±0.09	1.44 ±0.08	0.61 ±0.24	21.94 ±0.08
	**Mean[b]**	2.83 ±0.18	3.47 ±0.63	2.75 ±0.12	22.41 ±0.01	3.86 ±0.02	4.37 ±0.26	2.77 ± 0.05	22.65 ±0.23	3.18 ±0.18	3.57 ±0.32	2.91 ±0.47	22.42 ±0.07

[a] Standard deviation of the mean (SD); [b] Standard error of the mean (SE).

Table S4: Estimated ΔRMSF[a] parameter of ligands/M-pro proteins across stable structure trajectories (last 20 ns).

Sub-pocket	Residue	Compound 6 / M-pro protein residues				Compound 15 / M-pro protein residues				Lopinavir / M-pro protein residues			
		1st MD run	2nd MD run	3rd MD run	Mean ± SD	1st MD run	2nd MD run	3rd MD run	Mean ± SD	1st MD run	2nd MD run	3rd MD run	Mean ± SD
**S1′**	His41	**0.36**	**0.31**	**0.39**	**0.35 ±0.04**	-0.70	-0.69	-2.09	-1.16 ±0.81	**0.23**	**0.39**	**0.35**	**0.32 ±0.09**
	Gly143	-0.48	-0.35	-0.29	-0.37 ±0.10	0.06	-0.05	-0.14	-0.04 ±0.10	-0.37	-0.32	-0.91	-0.53 ±0.33
	Ser144	-0.19	-0.19	-0.12	-0.17 ±0.04	-0.21	-0.26	-0.27	-0.24 ±0.03	-0.18	-0.11	-0.24	-0.18 ±0.06
	Cys145	-0.07	-0.08	-0.01	-0.05 ±0.03	-0.33	-0.37	-0.37	-0.36 ±0.03	-0.09	-0.02	-0.16	-0.09 ±0.07
**S1**	Phe140	0.12	0.25	0.26	0.21 ±0.08	**0.52**	**0.44**	**0.50**	**0.49 ±0.04**	-0.06	0.04	-0.06	-0.03 ±0.06
	Leu141	-0.50	-0.20	-0.23	-0.31 ±0.16	0.28	0.14	0.21	0.21 ±0.07	-0.69	-0.53	-0.39	-0.53 ±0.15
	Asn142	-0.69	-0.44	-0.42	-0.52 ±0.15	0.11	-0.10	-0.02	0.001 ±0.10	-0.75	-0.74	-0.71	-0.73 ±0.02
	His163	**0.43**	**0.42**	**0.48**	**0.44 ±0.03**	-0.33	-0.84	-0.29	-0.48 ±0.31	**0.45**	**0.46**	**0.45**	**0.45 ±0.01**
	Glu166	**0.74**	**0.65**	**0.86**	**0.75 ±0.11**	**0.74**	**0.34**	**0.65**	**0.58 ±0.21**	**0.78**	**0.86**	**0.84**	**0.83 ±0.04**
**S2**	Met49	**1.24**	**1.45**	**1.96**	**1.55 ±0.37**	**1.71**	**1.83**	**1.64**	**1.73 ±0.09**	**1.33**	**1.30**	**1.00**	**1.21 ±0.18**
	Tyr54	**0.48**	**0.68**	**0.70**	**0.62 ±0.12**	0.23	**0.34**	0.29	0.29 ±0.05	**0.66**	**0.59**	**0.51**	**0.59 ±0.08**
	His164	**0.33**	**0.36**	**0.42**	**0.37 ±0.05**	-0.29	-0.91	-0.38	-0.53 ±0.34	**0.42**	**0.44**	**0.43**	**0.43 ±0.01**
	Asp187	**0.91**	**0.94**	**1.01**	**0.95 ±0.05**	**0.51**	**0.54**	**0.59**	**0.55 ±0.04**	**0.75**	**0.82**	**0.93**	**0.84 ±0.09**
	Arg188	**0.78**	**0.84**	**0.96**	**0.86 ±0.09**	**0.63**	**0.43**	**0.45**	**0.50 ±0.11**	**0.76**	**0.36**	**0.68**	**0.60 ±0.22**
**S3**	Met165	**0.31**	0.09	**0.42**	0.27 ±0.17	**0.01**	**-0.60**	**-0.13**	-0.24 ±0.32	**0.46**	**0.47**	**0.35**	**0.43 ±0.07**
	Leu167	**0.70**	**0.45**	**0.91**	**0.69 ±0.23**	**1.01**	**0.74**	**0.92**	**0.89 ±0.14**	**0.73**	**0.79**	**0.71**	**0.74 ±0.04**
	Gln189	**0.49**	**0.40**	**0.77**	**0.55 ±0.20**	**0.71**	**0.46**	**0.53**	**0.56 ±0.13**	-0.40	-0.03	**0.36**	-0.02 ±0.38
	Thr190	0.15	0.01	**0.47**	0.21 ±0.24	**0.35**	0.20	0.21	0.25 ±0.09	-1.46	-0.24	0.05	-0.55 ±0.80
	Gln192	0.02	0.00	0.32	0.11 ±0.18	-0.34	-0.06	0.06	-0.11 ±0.21	-1.84	-0.09	0.15	-0.59 ±1.09
**Vicinal residues**	Pro39	**0.49**	**0.57**	**0.60**	**0.55 ±0.06**	-0.48	0.01	-0.23	-0.23 ±0.25	**0.51**	**0.60**	**0.49**	**0.53 ±0.06**
	Val42	**1.14**	**1.09**	**1.19**	**1.14 ±0.05**	-0.06	0.01	-1.19	-0.41 ±0.67	**1.06**	**1.18**	**1.09**	**1.11 ±0.06**
	Ile43	**1.71**	**1.73**	**1.80**	**1.75 ±0.04**	**0.89**	**0.85**	**0.73**	**0.82 ±0.08**	**1.72**	**1.76**	**1.66**	**1.71 ±0.05**

Cys44	**2.14**	**2.15**	**2.42**	**2.24 ±0.16**	**1.68**	**2.13**	**1.36**	**1.72 ±0.38**	**2.22**	**2.37**	**2.18**	**2.25 ±0.10**
Thr45	**1.89**	**1.51**	**2.27**	**1.89 ±0.38**	**1.43**	**2.30**	**1.34**	**1.69 ±0.53**	**1.79**	**2.30**	**1.59**	**1.89 ±0.36**
Ser46	**1.52**	**1.20**	**2.10**	**1.60 ±0.46**	**0.79**	**2.29**	**1.38**	**1.49 ±0.76**	**1.84**	**1.68**	**1.36**	**1.62 ±0.24**
Glu47	**2.04**	**2.36**	**2.76**	**2.39 ±0.36**	**1.69**	**3.12**	**1.33**	**2.05 ±0.95**	**1.71**	**1.99**	**1.98**	**1.89 ±0.16**
Asp48	**1.80**	**1.44**	**2.41**	**1.89 ±0.49**	**1.81**	**2.12**	**1.86**	**1.93 ±0.17**	**0.71**	**1.63**	**1.17**	**1.17 ±0.46**
Leu50	**0.69**	**1.09**	**1.66**	**1.15 ±0.49**	**1.34**	**1.39**	**1.18**	**1.30 ±0.11**	**1.28**	**0.51**	**0.13**	**0.64 ±0.59**
Asn53	-0.03	0.53	0.06	0.18 ±0.30	**0.61**	**0.47**	**0.43**	**0.51 ±0.09**	**0.42**	0.00	-0.03	0.13 ±0.25
Pro168	0.05	-0.24	**0.45**	0.09 ±0.34	**0.75**	**0.68**	**0.68**	**0.70 ±0.04**	0.24	0.16	-0.03	0.13 ±0.14
Phe185	**0.36**	**0.40**	**0.52**	**0.43 ±0.08**	-0.51	0.05	-0.34	-0.27 ±0.29	0.27	**0.35**	**0.49**	**0.37 ±0.11**
Val186	**0.66**	**0.70**	**0.84**	**0.73 ±0.10**	-0.24	0.12	0.11	0.001 ±0.20	**0.55**	**0.49**	**0.74**	**0.59 ±0.13**

[a] Relative difference root-mean-square fluctuation (ΔRMSF) estimated for each ligand-bound protein relative to the apo state of COVID-19 M-pro. Only values with significant mobility changes (ΔRMSF > 0.3 Å) are represented in bold.

Table S5. Estimated RMSD and Rg parameter of investigated ligands and reference compound, Benzopurpurin 4B, complexed with Nsp15 NendoU proteins throughout the triplicate all-atom MD simulation

		**Compound 6 / Nsp15 NendoU complex**				**Compound 15 / Nsp15 NendoU complex**				**Benzopurpurin 4B / Nsp15 NendoU complex**			
		**RMSD protein** (Å)	**RMSD complex** (Å)	**RMSD ligand** (Å)	**Rg complex** (Å)	**RMSD protein** (Å)	**RMSD complex** (Å)	**RMSD ligand** (Å)	**Rg complex** (Å)	**RMSD protein** (Å)	**RMSD complex** (Å)	**RMSD ligand** (Å)	**Rg complex** (Å)
**1st MD run**	**Max.**	4.16	4.77	4.80	24.89	4.64	5.07	4.52	24.75	4.57	5.40	4.26	25.00
	**Min.**	1.45	1.72	1.83	23.39	2.02	2.01	1.54	23.21	1.85	1.86	0.98	23.40
	**Mean[a]**	2.61 ±0.32	3.55 ±0.41	3.59 ±0.32	23.931 ±0.20	2.79 ±0.30	4.01 ±0.37	3.55 ±0.36	23.80 ±0.20	2.69 ±0.31	4.00 ±0.42	2.98 ±0.47	23.96 ±0.19
**2nd MD run**	**Max.**	4.03	5.11	4.92	24.58	4.82	6.10	4.85	25.44	4.57	4.77	4.80	24.82
	**Min.**	1.69	1.79	1.83	23.40	1.64	1.89	2.11	23.41	1.67	1.67	0.96	23.36
	**Mean[a]**	2.55 ±0.27	3.00 ±0.42	4.07 ±0.45	23.87 ±0.19	2.82 ±0.36	4.29 ±0.51	3.32 ±0.41	24.02 ±0.31	2.63 ±0.36	2.83 ±0.36	2.94 ±0.32	23.84 ±0.18
**3rd MD run**	**Max.**	4.23	5.07	4.80	25.12	4.81	4.87	5.44	25.17	4.12	4.55	4.26	25.26
	**Min.**	1.79	2.01	1.76	23.32	1.89	2.01	1.59	23.35	1.66	1.82	1.00	23.42
	**Mean[a]**	2.72 ±0.37	4.01 ±0.37	3.59 ±0.32	23.89 ±0.25	2.65 ±0.41	3.95 ±0.31	4.12 ±0.44	24.01 ±0.30	2.70 ±0.35	3.41 ±0.28	2.98 ±0.47	23.92 ±0.24
						**Average ± SD/SE**							
	**Max.[a]**	4.14 ±0.10	4.98 ±0.18	4.84 ±0.07	24.86 ±0.27	4.76 ±0.10	5.34 ±0.66	4.94 ±0.46	25.12 ±0.35	4.42 ±0.26	4.91 ±0.44	4.44 ±0.31	25.03 ±0.22
	**Min.[a]**	1.64 ±0.17	1.84 ±0.15	1.81 ±0.04	23.37 ±0.04	1.85 ±0.19	1.97 ±0.07	1.75 ±0.32	23.32 ±0.10	1.73 ±0.11	1.87 ±0.10	0.50 ±0.03	23.39 ±0.03
	**Mean[b]**	2.62 ±0.19	3.52 ±0.23	3.75 ±0.21	23.90 ±0.12	2.75 ±0.21	4.08 ±0.23	3.66 ± 0.23	23.95 ±0.16	2.67 ±0.20	3.41 ±0.20	2.97 ±0.24	23.91 ±0.12

[a] Standard deviation of the mean (SD); [b] Standard error of the mean (SE).

Table S6: Estimated ΔRMSF[a] parameter of ligands/Nsp15 NendoU bound proteins across stable structure trajectories (last 20 ns).

**Sub-pocket**	**Residue**	**Compound 6 / Nsp15 NendoU protein residues**				**Compound 15 / M-pro protein residues**				**Lopinavir / M-pro protein residues**			
		**1st MD run**	**2nd MD run**	**3rd MD run**	**Mean ± SD**	**1st MD run**	**2nd MD run**	**3rd MD run**	**Mean ± SD**	**1st MD run**	**2nd MD run**	**3rd MD run**	**Mean ± SD**
**B0**	Asn278	0.09	0.12	-0.98	-0.26 ±0.62	-0.09	0.11	0.10	0.04 ±0.11	-0.02	-0.06	-0.03	-0.04 ±0.02
	Lys345	**1.53**	**0.74**	**0.88**	**1.05 ±0.42**	**1.21**	**1.19**	**1.58**	**1.32 ±0.22**	**0.71**	-0.03	**1.14**	**0.61 ±0.59**
	Leu346	**1.42**	**0.10**	**0.34**	**0.62 ±0.70**	**0.48**	**0.30**	**1.02**	**0.66 ±0.59**	**1.02**	**1.02**	**1.03**	**1.02 ±0.006**
**B1**	Gln245	-0.28	-0.18	-1.40	-0.62 ±0.68	-0.38	-0.21	-0.03	-0.21 ±0.18	0.11	-0.13	0.07	0.01 ±0.13
	Gly248	-0.06	-0.03	-0.53	-0.21 ±0.28	-0.07	-0.06	0.13	0.15 ±0.11	0.23	0.08	0.05	0.12 ±0.10
	His250	0.01	-0.01	-0.35	-0.12 ±0.20	-0.06	0.10	0.06	0.01 ±0.09	0.07	0.00	0.06	0.04 ±0.04
	Lys290	**0.44**	**0.31**	0.17	**0.31 ±0.14**	**0.37**	0.20	**0.39**	**0.32 ±0.10**	-0.01	-0.13	-0.03	-0.06 ±0.07
	Ser294	-0.09	0.02	-0.73	-0.27 ±0.40	-0.22	0.02	-0.05	-0.09 ±0.13	0.07	-0.05	0.01	0.01 ±0.06
	Tyr343	0.11	-0.09	-0.19	-0.06 ±0.15	0.02	-0.08	0.17	0.04 ±0.12	-0.04	-0.04	0.10	0.002 ±0.08
**B2**	His235	-0.17	-0.18	-0.62	-0.33 ±0.26	-0.28	-0.05	-0.14	-0.16 ±0.12	0.16	-0.01	0.01	0.05 ±0.09
	Gly247	-0.14	-0.07	-0.75	-0.32 ±0.38	-0.21	-0.03	0.16	-0.03 ±0.18	0.21	0.02	0.02	0.08 ±0.11
	Trp333	0.25	-0.04	-0.10	0.04 ±0.19	**0.33**	0.27	**0.38**	**0.33 ±0.06**	0.19	-0.03	0.22	0.12 ±0.14
	Thr341	-0.06	-0.20	-0.49	-0.25 ±0.22	-0.04	-0.05	0.08	-0.01 ±0.07	0.15	-0.04	0.11	0.07 ±0.10
**B3**	Asp240	**0.65**	**0.71**	0.13	**0.50 ±0.32**	**0.68**	**0.77**	**0.89**	**0.78 ±0.11**	**0.72**	**0.53**	**0.65**	**0.63 ±0.10**
	His243	**0.52**	**0.62**	0.24	**0.46 ±0.20**	**0.52**	**0.62**	**0.79**	**0.64 ±0.13**	**0.72**	**0.49**	**0.71**	**0.64 ±0.13**
	Glu340	-0.09	-0.18	-0.64	-0.03 ±0.30	-0.02	-0.05	0.12	0.01 ±0.09	0.19	0.00	0.18	0.13 ±0.11
**Vicinal residues**	Leu215	**0.39**	**0.48**	**0.36**	**0.41 ±0.06**	0.28	**0.42**	**0.45**	**0.38 ±0.09**	0.21	0.13	0.20	0.18 ±0.05
	Ile223	**0.47**	**0.61**	0.21	**0.43 ±0.21**	**0.40**	**0.51**	**0.52**	**0.48 ±0.07**	0.23	0.18	0.19	0.20 ±0.03
	Tyr226	0.28	**0.40**	0.05	0.24 ±0.18	0.27	**0.33**	**0.35**	**0.31 ±0.04**	0.17	0.16	0.17	0.17 ±0.01
	Leu228	0.23	**0.43**	0.06	0.24 ±0.18	0.29	**0.38**	**0.38**	**0.35 ±0.05**	0.22	0.17	0.11	0.17 ±0.05
	Gly239	**0.34**	**0.31**	-0.09	0.19 ±0.24	0.29	**0.41**	**0.48**	**0.39 ±0.10**	**0.36**	0.16	0.26	0.26 ±0.10

Phe241	**1.07**	**1.17**	0.21	**0.82 ±0.53**	**1.14**	**1.20**	**1.36**	**1.23 ±0.11**	**1.14**	**0.91**	**1.05**	**1.03 ±0.12**
Ser242	**0.77**	**0.89**	-0.28	**0.46 ±0.64**	**0.83**	**0.86**	**1.04**	**0.91 ±0.11**	**0.94**	**0.74**	**0.94**	**0.87 ±0.11**
Leu332	0.20	-0.01	0.11	0.10 ±0.10	**0.32**	**0.30**	**0.36**	**0.33 ±0.03**	0.21	-0.06	0.22	0.13 ±0.16
Cys334	**0.50**	0.24	0.01	0.25 ±0.24	**0.58**	**0.41**	**0.64**	**0.54 ±0.12**	0.24	-0.05	0.27	0.15 ±0.18
Lys335	0.23	0.01	-0.61	-0.12 ±0.43	0.29	0.05	**0.34**	0.23 ±0.16	0.26	-0.03	0.36	0.19 ±0.20

[a] Relative difference root-mean-square fluctuation (ΔRMSF) estimated for each ligand-bound protein relative to the apo state of COVID-19 Nsp15 NendoU. Only values with significant mobility changes (ΔRMSF > 0.3 Å) are represented in bold.

Table S7. Total binding-free energies and individual energy term (ΔG ± SD) of the promising scalarane-based compounds and reference ligands within Nsp15 NendoU and/or M-pro protein binding site, across the three MD simulation runs.

	**Energy** (kJ/mol ± SD)	**M-pro complex**			**Nsp15 NendoU complex**		
		**Comp. 6**	**Comp. 15**	**Lopinavir**	**Comp. 6**	**Comp. 15**	**Benzopurpurin 4B**
**1st MD run**	**van der Waal**	-110.60 ± 18.86	-119.83 ± 11.25	-215.43 ± 19.69	-102.33 ± 14.27	-80.21 ± 11.17	-117.31 ± 20.19
	**Electrostatic**	-13.65 ± 26.693	-13.82 ± 18.22	-26.62 ± 20.49	-46.560 ± 13.84	-8.39 ± 17.01	-82.22 ± 33.47
	**Solvation; Polar**	70.06 ± 32.18	85.87 ± 23.47	160.01 ± 35.13	115.28 ± 24.61	26.98 ± 35.32	143.16 ± 42.38
	**Solvation; SASA**	-13.50 ± 1.49	-14.76 ± 1.44	-21.76 ± 1.83	-12.78 ± 1.20	-11.80 ± 1.80	-17.89 ± 2.87
	**Binding energy**	-67.69 ± 16.45	-62.53 ± 12.37	-103.81 ± 31.62	-46.43 ± 27.21	-73.42 ± 25.24	-74.26 ± 34.28
**2nd MD run**	**van der Waal**	-149.98 ± 21.32	-105.14 ± 22.53	-178.95 ± 33.26	-95.76 ± 15.75	-75.83 ± 32.75	-105.49 ± 21.29
	**Electrostatic**	-6.94 ± 10.30	-20.54 ± 19.49	-32.07 ± 14.69	-56.13 ± 8.54	-10.13 ± 4.11	-112.62 ± 4.91
	**Solvation; Polar**	87.58 ± 11.60	62.37 ± 35.58	146.35 ± 28.35	105.52 ± 14.81	16.20 ± 51.13	153.72 ± 44.05
	**Solvation; SASA**	-16.28 ± 1.04	-11.12 ± 3.01	-19.69 ± 2.84	-12.75 ± 1.71	-6.65 ± 3.33	-14.05 ± 2.22
	**Binding energy**	-85.63 ± 16.05	-74.44 ± 19.91	-84.36 ± 21.68	-59.12 ± 14.68	-76.41 ± 43.76	-78.44 ± 22.75
**3rd MD run**	**van der Waal**	-138.19 ± 14.81	-95.14 ± 22.06	-174.33 ± 12.35	-98.08 ± 10.69	-99.74 ± 18.93	-110.28 ± 34.65
	**Electrostatic**	-7.46 ± 12.43	-8.81 ± 10.97	-35.98 ± 14.71	-53.16 ± 18.98	-4.63 ± 6.50	-112.53 ± 28.68
	**Solvation; Polar**	86.31 ± 10.14	43.24 ± 35.17	140.20 ± 20.91	120.34 ± 27.70	32.90 ± 50.22	173.66 ± 42.55
	**Solvation; SASA**	-16.46 ± 1.46	-7.40 ± 3.19	-20.51 ± 1.09	-12.74 ± 1.25	-12.31 ± 1.88	-16.62 ± 3.53
	**Binding energy**	-75.79 ± 17.85	-68.11 ± 37.80	-90.62 ± 21.23	-43.65 ± 18.04	-83.78 ± 32.28	-65.78 ± 35. 29

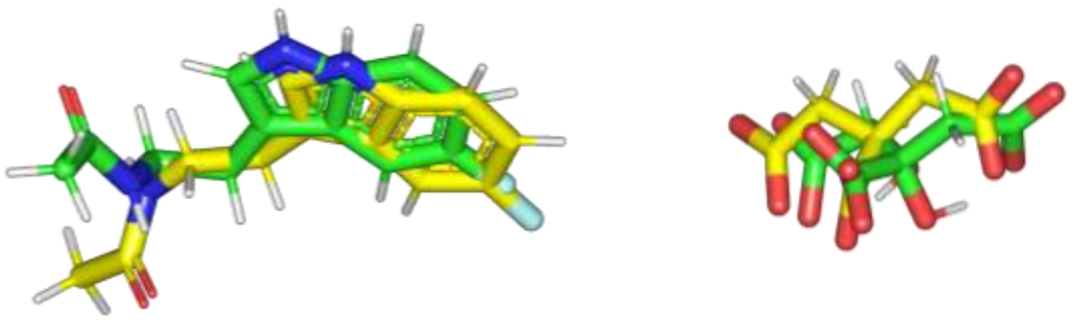

(A) **(B)**

Figure S1. Superimposing the crystallized (green sticks) and redocked (yellow sticks) ligands at **(A)** M-pro and (**B)** Nsp15 NendoU for validating the adopted directed docking protocol.

.

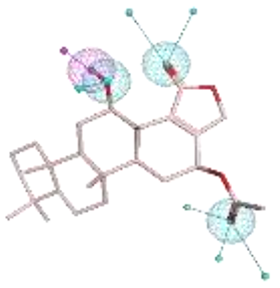

Compound 1

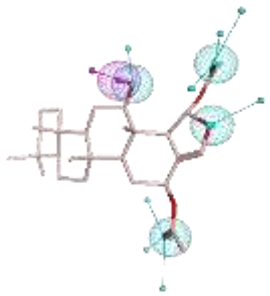

Compound 2

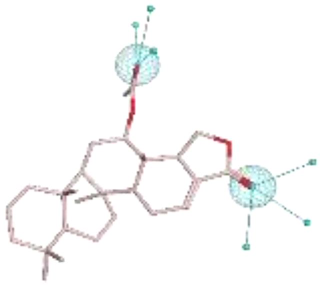

Compound 4

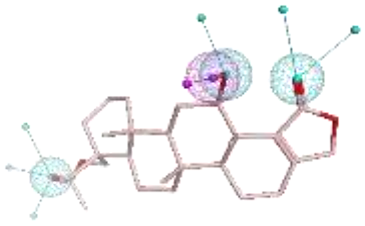

Compound 6

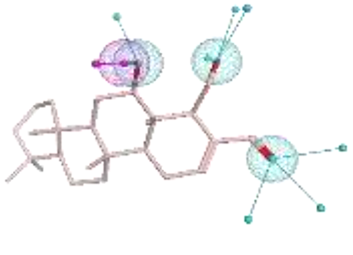

Compound 8

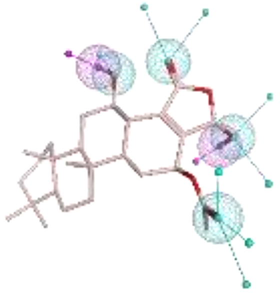

Compound 10

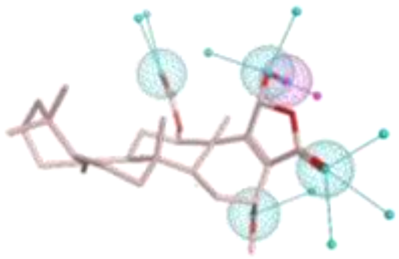

Compound 11

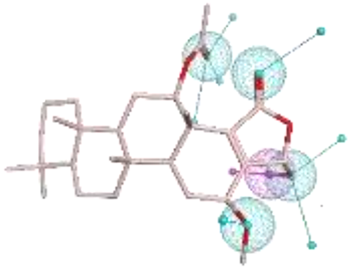

Compound 12

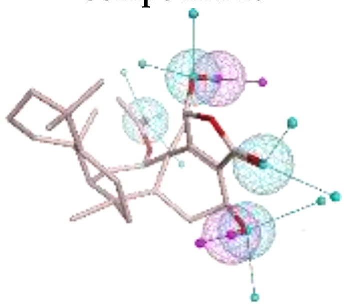

Compound 15

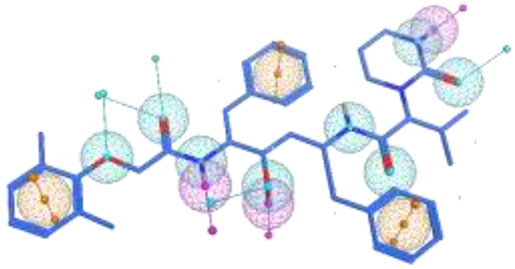

Lopinavir

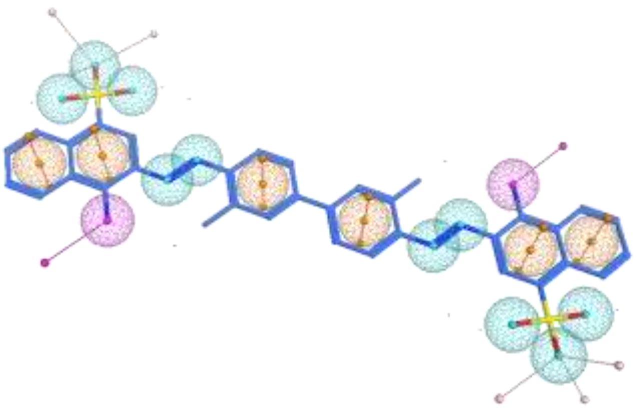

Benzopurpurin 4B

Figure S2. The 3D structural representation of the promising scalarane-based hits (sticks) and reference ligands showing respective pharmacophoric features (mesh spheres) as well as projected virtual points (arrows). Features and directionality of H-bond donor, H-bond acceptor, or aromaticity are colored in violet, cyan, or orange, respectively.
